# FIONA1‐Mediated m^6^A Modification Regulates the Floral Transition in *Arabidopsis*

**DOI:** 10.1002/advs.202103628

**Published:** 2022-01-05

**Authors:** Tao Xu, Xiaowei Wu, Chui Eng Wong, Sheng Fan, Yu Zhang, Songyao Zhang, Zhe Liang, Hao Yu, Lisha Shen

**Affiliations:** ^1^ Department of Biological Sciences National University of Singapore 14 Science Drive 4 Singapore 117543 Singapore; ^2^ Temasek Life Sciences Laboratory National University of Singapore 1 Research Link Singapore 117604 Singapore; ^3^ Biotechnology Research Institute Chinese Academy of Agricultural Sciences Beijing 100081 China

**Keywords:** *Arabidopsis*, FIO1, flowering, m^6^A, m^6^A writers

## Abstract

*N* ^6^‐methyladenosine (m^6^A) mRNA modification represents the most widespread form of internal modifications in eukaryotic mRNAs. In the model plant *Arabidopsis thaliana*, those known methyltransferases mainly deposit m^6^A at their target transcripts near the stop codon or in the 3′ untranslated region. Here, it is reported that FIONA1 (FIO1), a human METTL16 ortholog, acts as a hitherto unknown m^6^A methyltransferase that determines m^6^A modifications at over 2000 *Arabidopsis* transcripts predominantly in the coding region. Mutants of *FIO1* show a decrease in global m^6^A mRNA methylation levels and an early‐flowering phenotype. Nanopore direct RNA sequencing reveals that FIO1 is required for establishing appropriate levels of m^6^A preferentially in the coding sequences of a subset of protein‐coding transcripts, which is associated with changes in transcript abundance and alternative polyadenylation. It is further demonstrated that FIO1‐mediated m^6^A methylation determines the mRNA abundance of a central flowering integrator *SUPPRESSOR OF OVEREXPRESSION OF CONSTANS 1* (*SOC1*) and its upstream regulators, thus preventing premature flowering. The findings reveal that FIO1 acts as a unique m^6^A methyltransferase that mainly modifies the coding regions of transcripts, which underlies the key developmental transition from vegetative to reproductive growth in plants.

## Graphical abstract

This work uncovers the function of FIONA1 as a unique m^6^A methyltransferase that mainly modifies the coding regions of transcripts, including those flowering‐related transcripts, to determine the transition from vegetative to reproductive development in plants. This study provides novel insights into understanding of m^6^A landscape, the associated new players, and their biological functions in plants.

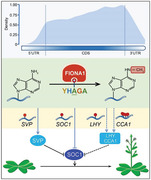

## 1 Introduction

*N* ^6^‐methyladenosine (m^6^A), the most prevalent internal modification in mRNAs found in many eukaryotes, has emerged as a key regulatory mechanism to control gene expression. m^6^A modifications could serve as key switches on mRNA metabolism through affecting splicing, stability, alternative polyadenylation, secondary structure, nuclear export, and translation.^[^ ^1^, ^2^, ^3^, ^4^ ^]^ Transcriptome‐wide profiling of m^6^A modifications is thus fundamental for decoding the genome and can be mapped with the next generation sequencing, such as the antibody‐based m^6^A‐seq and m^6^A‐CLIP approaches,^[^ ^5^, ^6^ ^]^ and the third generation sequencing by Oxford nanopore technology.^[^ ^7^ ^]^ Notably, nanopore technology that is able to directly sequence native full‐length RNA molecules overcomes the limitations of next generation sequencing that is based on short‐read cDNA sequencing and demands the conversion of RNA to cDNA.^[^ ^8^, ^9^ ^]^ In nanopore direct RNA sequencing, RNA sequences can be identified by the magnitudes of electric intensity across the nanopore surface when RNA passes the pore. RNA modifications cause shifts in the intensity levels so that the modified bases can be computationally identified at the single‐base resolution.^[^ ^8^, ^9^, ^10^, ^11^ ^]^

m^6^A is a reversible modification and is dynamically regulated by the concerted cooperation of methyltransferases (writers), demethylases (erasers), and m^6^A binding protein (readers) that install, remove, and interpret m^6^A, respectively. In *Arabidopsis thaliana*, m^6^A methylation is deposited by a multicomponent methyltransferase complex containing mRNA adenosine methylase (MTA), MTB, FKBP12 INTERACTING PROTEIN 37KD (FIP37), VIRILIZER (VIR), and HAKAI.^[^ ^12^, ^13^, ^14^, ^15^ ^]^ This methyltransferase complex methylates thousands of transcripts mainly at regions near the stop codon and in the 3′ untranslated region (3′ UTR), and preferentially in the RRACH (R = A/G; H = A/C/U) motif.^[^ ^9^, ^13^ ^]^ Disruption of the component genes of the m^6^A methyltransferase complex, including MTA, FIP37, and VIR, results in greatly reduced (≈80–90% reduction), but not completely abolished m^6^A modifications.^[^ ^12^, ^13^, ^16^ ^]^ Moreover, a transcriptome‐wide mapping of m^6^A sites in *fip37* mutants (*fip37‐4 LEC1:FIP37*) has revealed that loss of function of *FIP37* mainly affects the m^6^A peaks near the stop codon and 3′ UTR, but has less impact on those in the coding sequence (CDS) and 5′ UTR.^[^ ^13^ ^]^ These findings indicate the presence of other unknown m^6^A methyltransferase(s) in *Arabidopsis*.

In this study, we show that FIONA1 (FIO1) acts as a hitherto unknown m^6^A methyltransferase in *Arabidopsis*. FIO1 is a nucleus‐localized protein^[^ ^17^ ^]^ and orthologous to the human METTL16 that installs m^6^A on diverse RNA molecules, such as U6 small nuclear RNA (snRNA), the *S*‐adenosylmethionine (SAM) synthetase *MAT2A* pre‐mRNA, and possibly other RNAs.^[^ ^18^, ^19^, ^20^ ^]^ Both U6 snRNA and *MTA2A* contain a conserved sequence, UACm ^6^ AGAGAA, required for METTL16‐mediated methylation. Here, we show that FIO1 functions as an m^6^A methyltransferase that is responsible for establishing appropriate levels of m^6^A modifications on a subset of protein‐coding transcripts mainly in the CDS in *Arabidopsis*. Disruption of *FIO1* results in early flowering and a mild decrease in global m^6^A levels. Nanopore direct RNA sequencing uncovers a total of 3459 high‐confidence hypomethylated m^6^A sites in 2068 protein‐coding genes, including the key flowering time integrator *SUPPRESSOR OF OVEREXPRESSION OF CONSTANS 1* (*SOC1*) and its upstream genes. Our findings reveal the role of FIO1 as a novel m^6^A methyltransferase that underlies the control of the floral transition in plants.

## 2 Results and Discussion

### 2.1 Disruption of *FIO1* Results in a Mild Reduction in Global mRNA m^6^A Levels

**Figure 1:**
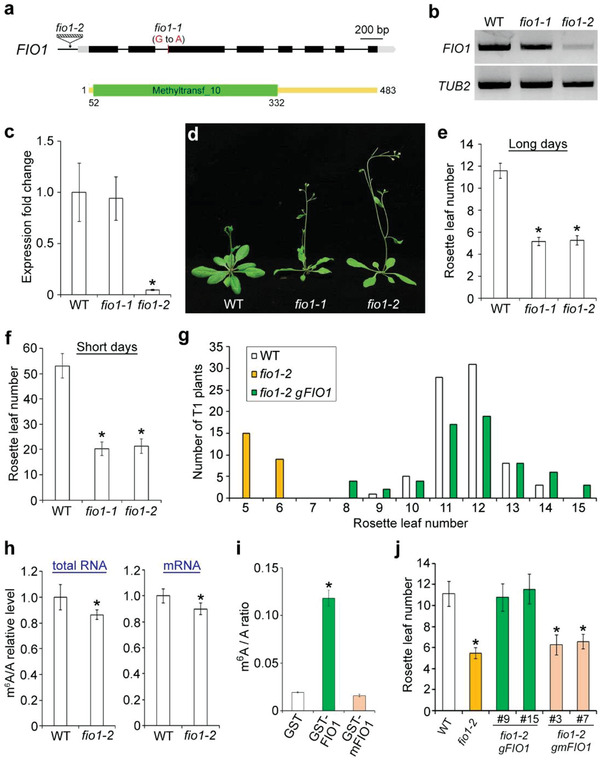
FIO1 affects flowering and mRNA m^6^A levels in *Arabidopsis*. a) Schematic diagrams show the mutation site in *fio1‐1* and the T‐DNA insertion site in *fio1‐2* (upper panel) and the methyltransferase domain (green box) in the FIO1 protein (lower panel). Exons in coding sequence and untranslated regions (UTRs) are shown in black and gray boxes, respectively, while introns and other genomic sequences are shown in black lines. *fio1‐1* contains a G to A conversion in the last nucleotide in the second intron. b) Semiquantitative RT‐PCR shows the expression of *FIO1* in *fio1* mutants. *TUB2* expression was used an internal control. c) Quantitative real‐time PCR analysis of *FIO1* expression in 6‐day‐old seedlings of various genetic background. The expression level of *FIO1* in wild‐type seedlings was set as 1.0. Error bars, mean ± SD; *n* = 3 biological replicates. Asterisk indicates a significant difference between *fio1‐2* and wild‐type seedlings (two‐tailed paired Student's *t*‐test, *P* < 0.001). d) Loss of *FIO1* greatly accelerates flowering under long days. Flowering time of *fio1‐1* and *fio1‐2* grown under e) long days and f) short days. Error bars, mean ± SD; *n* = 20. Asterisks indicate significant differences between *fio1* mutants and wild‐type plants (two‐tailed paired Student's *t*‐test, *P* < 0.001). g) Flowering time distribution of T1 transgenic plants of *fio1‐2 gFIO1*. The flowering time of each individual T1 transgenic lines of *fio1‐2 gFIO1* was scored. h) Measurement of m^6^A level relative to that of adenosine (m^6^A/A) by LC‐MS/MS in total RNA (left panel) and mRNA (right panel) isolated from 6‐day‐old wild‐type and *fio1‐2* seedlings. The m^6^A/A ratios in wild‐type seedlings were set as 1.0. Error bars, mean ± SD; *n* = 3 biological replicates × 3 technical replicates. Asterisks indicate significant differences between *fio1‐2* and wild‐type seedlings (two‐tailed paired Student's *t*‐test, *P* < 0.01). i) Measurement of m^6^A level relative to that of adenosine (m^6^A/A) by LC‐MS/MS in RNA purified from the m^6^A methylation assay. RNA oligo (GCCAGAGCCAGAGCCAGAGCCAGA) containing four repeats of the consensus m^6^A motif recognized by FIO1 was incubated with GST, GST‐FIO1, and GST‐mFIO1, after which RNA was purified for measurement of m^6^A levels by LC‐MS/MS analysis. Error bars, mean ± SD; *n* = 3 biological replicates. Asterisk indicates a significant difference between GST‐FIO1 and GST or GST‐mFIO1 (two‐tailed paired Student's *t*‐test, *P* < 0.01). j) Flowering time of representative *fio1‐2 gFIO1* lines (9 and 15) and *fio1‐2 gmFIO1* lines (3 and 7) grown under long days. Error bars, means ± SD; *n* = 20. Asterisks indicate significant differences between the specified genotypes and wild‐type plants (two‐tailed paired Student's *t*‐test, *P* < 0.01).

FIO1 contains a methyltransferase domain (**Figure** **1**a) and is the only *Arabidopsis* ortholog of the human METTL16 (Figure S1, Supporting Information). Within the methyltransferase domain, FIO1 contains the key catalytic motif NPPF (residues 236–239), which is highly conserved among FIO1 homologs in various organisms (Figure S1, Supporting Information). *FIO1* was widely expressed in various *Arabidopsis* tissues (Figure S2, Supporting Information). To study whether FIO1 is involved in m^6^A methylation in *Arabidopsis*, we isolated two *fio1* mutants, *fio1‐1* containing a G to A conversion at the splice acceptor site of the 2nd intron^[^ ^17^ ^]^ and *fio1‐2* carrying a T‐DNA insertion in the 5′ upstream region (Figure 1a; Figure S3a, Supporting Information). The mutation in *fio1‐1* resulted in a 5 amino acid deletion of FIO1 protein sequence (Figure S3b, Supporting Information), but did not affect the *FIO1* mRNA expression, whereas in *fio1‐2* mutants, *FIO1* expression was greatly reduced (Figure 1b,c). Both *fio1‐1* and *fio1‐2* exhibited early flowering phenotypes under long days (Figure 1d,e) and short days (Figure 1f). Since *fio1‐1* and *fio1‐2* showed a similar flower time defect, we used *fio1‐2* for further studies. A genomic fragment of *FIO1* (*gFIO1*) fully rescued the early flowering phenotype of *fio1‐2* (Figure 1g; Figure S4, Supporting Information), demonstrating that *FIO1* is responsible for the early‐flowering phenotype observed in *fio1‐2*.

We then examined m^6^A levels in total RNAs isolated from 6‐day‐old wild‐type and *fio1‐2* seedlings by dot blot analysis using anti‐m^6^A antibody and found a slight reduction of m^6^A levels in *fio1‐2* (Figure S5, Supporting Information). Further quantitative measurement of m^6^A levels by liquid chromatography‐tandem mass spectrometry (LC‐MS/MS) revealed that m^6^A levels of total RNA and mRNA in *fio1‐2* were decreased by ≈14% and ≈10%, respectively, as compared with those in wild‐type seedlings (Figure 1h). These results suggest that *FIO1* is involved in m^6^A methylation in *Arabidopsis*. Expression levels of known m^6^A writer genes, such as *MTA*, *MTB*, *FIP37*, *HAKAI*, and *VIR*,^[^ ^12^, ^13^, ^14^ ^]^ as well as the m^6^A eraser gene *ALKBH10B* ^[^ ^21^ ^]^ remained unchanged in *fio1‐2* (Figure S6a, Supporting Information), suggesting that FIO1 may be directly involved in depositing m^6^A.

### 2.2 Nanopore Direct RNA Sequencing Identified Hypomethylated Sites in CDSs in *fio1* Mutants

To reveal how FIO1 contributes to the global m^6^A mRNA modification, we performed nanopore direct RNA sequencing on poly(A)‐tailed mRNAs from three biological replicates of 6‐day‐old wild‐type and *fio1‐2* seedlings. We obtained around three and two million of high‐quality reads (*Q*‐score > 7) for wild‐type and *fio1‐2* seedlings, respectively. Most of the reads are of high‐quality with the *Q*‐score of around 11 and an average read length of 912–945 nt for each library (Figure S7a,b and Table S1, Supporting Information). This is in line with a previous study showing an average read length of 900–1000 nt of *Arabidopsis* mRNA.^[^ ^22^ ^]^ In addition, we also observed long reads over 11600 nt (Figure S7c, Supporting Information). These observations indicate high integrity of our nanopore reads that can be used for subsequent analyses.

**Figure 2:**
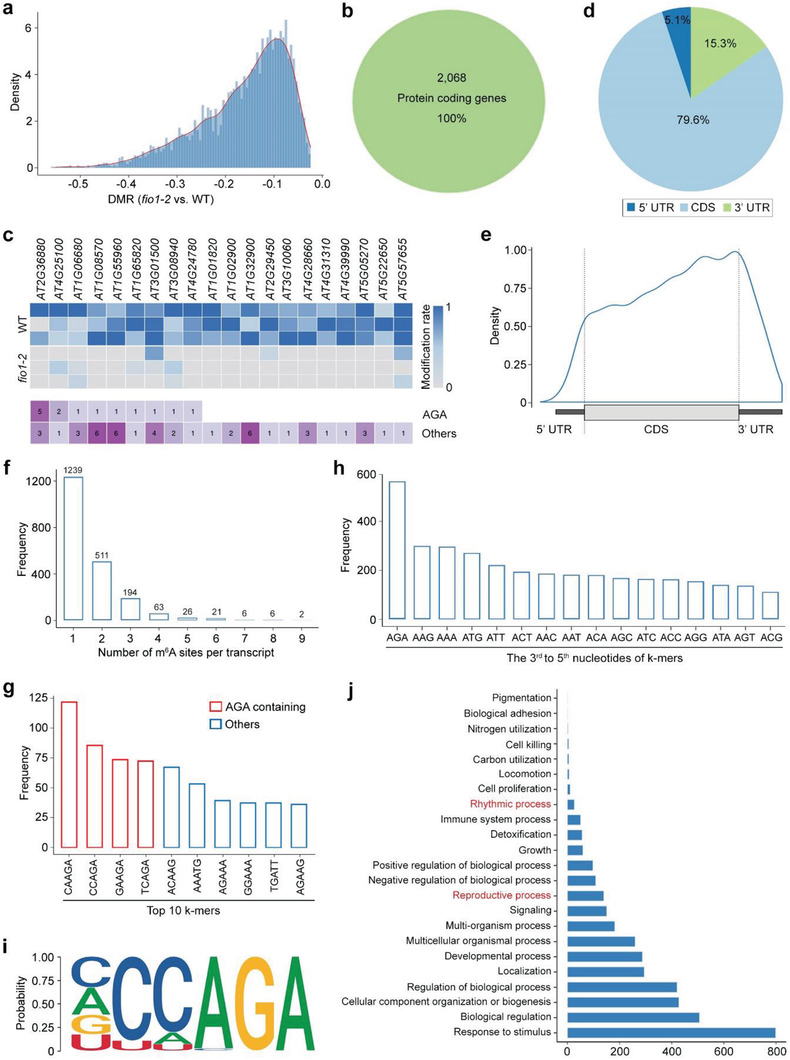
Distribution of hypomethylated sites in *fio1‐2*. a) Distribution of DMR for hypomethylated sites in *fio1‐2*. DMR represents the difference between the modification rates detected between wild‐type and *fio1‐2* plants. b) The hypomethylated sites are in 2068 protein‐coding transcripts. c) Heatmap showing the modification rates of top 20 significantly hypomethylated sites in *fio1‐2* ranked by *P*‐value (upper panel) and number of modification sites with AGA or others (lower panel). d) The pie chart displaying the percentages of hypomethylated sites in *fio1‐2* in different segments of transcripts divided into 5′ UTR, CDS, and 3′ UTR. e) Distribution of hypomethylated sites in *fio1‐2* along the transcript divided into 5′ UTR, CDS, and 3′ UTR. f) Frequency of numbers of m^6^A sites per transcript of hypomethylated genes in *fio1‐2*. g) Frequency of the top 10 5‐bp k‐mers at the positions with significantly differential modification rates between wild‐type and *fio1‐2* plants. h) Frequency of the last 3 nucleotides of k‐mers at the positions with significantly differential modification rates between wild‐type and *fio1‐2* plants. i) Sequence logo representing the consensus motif (YHAGA) found in the hypomethylated sites in *fio1‐2*. “Y” represents C/U (C > U) and “H” represents C/A/U (C > A/U). j) Gene ontology (GO) enrichment analysis of hypomethylated genes in *fio1‐2*.

We first mapped the nanopore reads to the transcriptome by Minimap2,^[^ ^23^ ^]^ and 96.6–98.4% of the reads were successfully mapped to TAIR10. After the signal segmentation with the Nanopolish software,^[^ ^24^ ^]^ we applied the xPore method^[^ ^8^ ^]^ to identify differential m^6^A RNA modifications between wild‐type and *fio1‐2*. xPore is a computational method that identifies positions of m^6^A modifications at the single‐base resolution and determines the differential modification rates across different conditions with high accuracy, and has been successfully used for profiling differential m^6^A modifications in human cell lines and cancer tissues.^[^ ^8^ ^]^ By calculating differential modification rates (DMRs) that represent differences between the modification rates in wild‐type and *fio1‐2* using A‐centered k‐mers (NNANN) with xPore, we identified a total of 3459 high‐confidence hypomethylated m^6^A sites that were consistently detected in all three biological replicates in 2068 protein‐coding genes in *fio1‐2* (*P* < 0.01; **Figure** **2**a,b; Table S2, Supporting Information). Most DMRs of hypomethylated sites were less than 50% (Figure 2a), which is in accordance with the mild decrease of total m^6^A levels observed in *fio1‐2* (Figure 1h). We ranked hypomethylated sites by the lowest *P* value and found that DMRs were consistent in each biological replicate (Figure 2c). After comparing the distribution of these hypomethylated sites along transcripts relative to landmarks in their architecture, we revealed that the majority (79.6%) of hypomethylated sites were enriched in the CDSs and peaked before the stop codon (Figure 2d,e). This is in contrast to the known m^6^A writers including FIP37 and VIR, which affect m^6^A modifications mainly near the stop codon and 3′ UTR.^[^ ^9^, ^13^ ^]^ Thus, FIO1‐dependent m^6^A sites constitute a distinct subset of m^6^A modifications in the CDSs. Interestingly, the hypomethylated sites revealed in this study were partially overlapped with the m^6^A sites identified by the other two antibody‐based sequencing approaches, m^6^A‐seq^[^ ^21^ ^]^ and miCLIP,^[^ ^9^ ^]^ using 14‐day‐old seedlings (Figure S8, Supporting Information). Whether this indicates different detection thresholds of various sequencing approaches or a dynamic feature of m^6^A modifications at different developmental stages needs to be further investigated.

Most of the hypomethylated genes contain only one hypomethylated m^6^A site (Figure 2f). Some of the top ranked sites containing genes by lowest *P* value appeared to contain multiple m^6^A sites involving different k‐mers (Figure 2c). The top four k‐mers in the positions with significantly reduced DMRs between wild‐type and *fio1‐2* plants were CAm ^6^ AGA, CCm ^6^ AGA, GAm ^6^ AGA, and TCm ^6^ AGA, which all contained the sequence of m ^6^ AGA at the last three nucleotides (Figure 2g). Consistently, m ^6^ AGA was observed as the most frequently occurred three‐nucleotide sequence in the 3rd to 5th positions of k‐mers (Figure 2h). Furthermore, we identified the YHm ^6^ AGA (Y = C/U; H = C/A/U) sequence as the most enriched motif among the hypomethylated sites using the HOMER program^[^ ^25^ ^]^ (*P* = 1e‐20; Figure 2i), implying that FIO1 preferentially targets to this motif for m^6^A methylation. Interestingly, the conserved sequence recognized by METTL16, UACm ^6^ AGAGAA,^[^ ^20^ ^]^ also contains an m ^6^ AGA sequence in the center. Further gene ontology (GO) analysis showed that the hypomethylated genes could regulate multiple biological processes (Figure 2j). Notably, the genes involved in plant reproductive process and rhythmic process were enriched, which could be associated with the early‐flowering (Figure 1) and lengthening of the free‐running circadian period phenotypes of *fio1* mutants.^[^ ^17^ ^]^ Taken together, these data demonstrate that FIO1 is responsible for m^6^A methylation on protein‐coding transcripts preferentially in the coding sequences in *Arabidopsis*.

To further examine whether FIO1 possesses the m^6^A methyltransferase activity, we performed in vitro methylation assay through incubating an RNA oligo containing the identified YHAGA motif (Figure 2i) with GST or the recombinant GST‐FIO1 protein, followed by examination of m^6^A levels by LC‐MS/MS and dot blot assays. GST‐FIO1, but not GST, methylates the RNA oligo (Figure 1i; Figure S9, Supporting Information), suggesting that FIO1 possesses the m^6^A methyltransferase activity. We then generated a catalytically inactive version of FIO1 by mutating the key catalytic residues NPPF to NAAF (mFIO1^237A 238A^; hereafter called mFIO1). Both LC‐MS/MS and dot blot assays revealed that mFIO1 lost the m^6^A methyltransferase activity (Figure 1i; Figure S9, Supporting Information). Moreover, *gmFIO1*, in which *mFIO1* was driven by the same *FIO1* promoter used in *gFIO1* for the gene complementation assay (Figure 1g), failed to rescue the early‐flowering phenotype of *fio1‐2* (Figure 1j), indicating that the m^6^A methyltransferase activity of FIO1 is essential for its function in flowering time regulation.

### 2.3 FIO1‐Mediated m^6^A Methylation Is Associated with Transcript Abundance and APA

**Figure 3:**
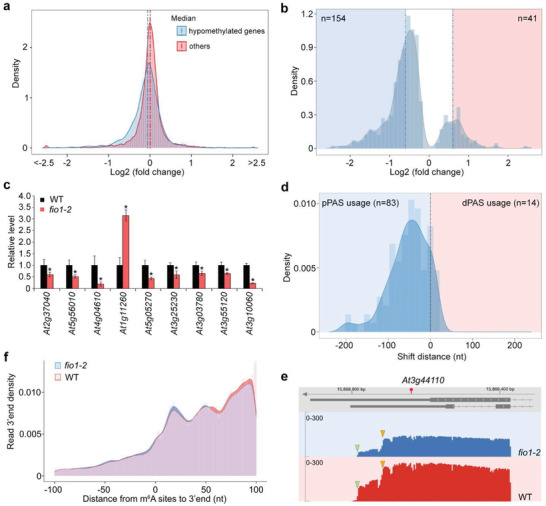
FIO1 regulates transcript abundance and alternative polyadenylation. a) Distribution of changes in gene expression between *fio1‐2* and wild‐type plants for hypomethylated genes and other genes (*P* < 10 × 10^−16^, two sided Mann–Whitney test). b) Distribution of differentially expressed genes with hypomethylated sites in *fio1‐2* compared with wild‐type seedlings (*P* < 0.05; log2 (fold change) > 0.6). c) Expression of several randomly chosen differentially expressed genes in *fio1‐2* determined by real‐time PCR. Six‐day‐old wild‐type and *fio1‐2* seedlings grown under long days were harvested for expression analysis. The expression levels of each gene in wild‐type seedlings were set as 1.0. Error bars, mean ± SD; *n* = 3 biological replicates. Asterisks indicate significant differences between *fio1‐2* and wild‐type seedlings (two‐tailed paired Student's *t*‐test, *P* < 0.05). d) A shift to the usage of proximal 3′ end polyadenylation sites found in *fio1‐2* compared with wild‐type. e) *At3g44110*, which is methylated in the 3′ UTR, shows a shift to the usage of the proximal polyadenylation site in *fio1‐2*. The position of the hypomethylated site is indicated by a red circle. Green and yellow triangles indicate the distal and proximal sites, respectively. f) Histogram showing the distance from the hypomethylated sites to the 3′ end of nanopore reads.

To investigate whether there is a potential correlation between m^6^A modification and gene expression levels mediated by FIO1, we identified differentially expressed genes (DEGs) between wild‐type and *fio1‐2* using the nanopore reads containing both RNA modifications and transcript processing information. There were 367 downregulated and 208 upregulated genes, respectively, in *fio1‐2* mutants (fold change >1.5; padj < 0.05) (Table S3, Supporting Information). Genes with hypomethylated sites tended to be downregulated in *fio1‐2* (**Figure** **3**a), and comparison of differentially expressed genes with the hypomethylated gene list revealed that the transcript abundance for 154 or 41 genes was decreased or increased in *fio1‐2*, respectively (Figure 3b). We confirmed the changes in transcript levels of several randomly selected genes (Figure 3c). These observations suggest that FIO1‐mediated m^6^A modification modulates transcript abundance in *Arabidopsis*. We further examined whether FIO1 affects pre‐RNA processing events. FIO1 had only a mild effect on RNA splicing and poly(A) tail length (Figures S10 and S11 and Table S4, Supporting Information). In addition, there were 15.3% of the hypomethylated sites located in the 3′ UTR (Figure 2d). Since m^6^A methylation in 3′ UTR is associated with 3′ end formation,^[^ ^9^ ^]^ we further examined whether FIO1 also affects the selection of alternative polyadenylation (APA) sites. Among 83 out of 97 transcripts shifting to the usage of proximal poly(A) sites (Figure 3d,e), 60.8% of these transcripts contained hypomethylated sites (Table S5, Supporting Information). The changes were mainly located downstream to the m^6^A sites (Figure 3f). Taken together, these data suggest that FIO1‐mediated m^6^A methylation is associated with transcript abundance and APA.

### 2.4 FIO1‐Mediated m^6^A Modification Suppresses *SOC1* Expression to Control Flowering

**Figure 4:**
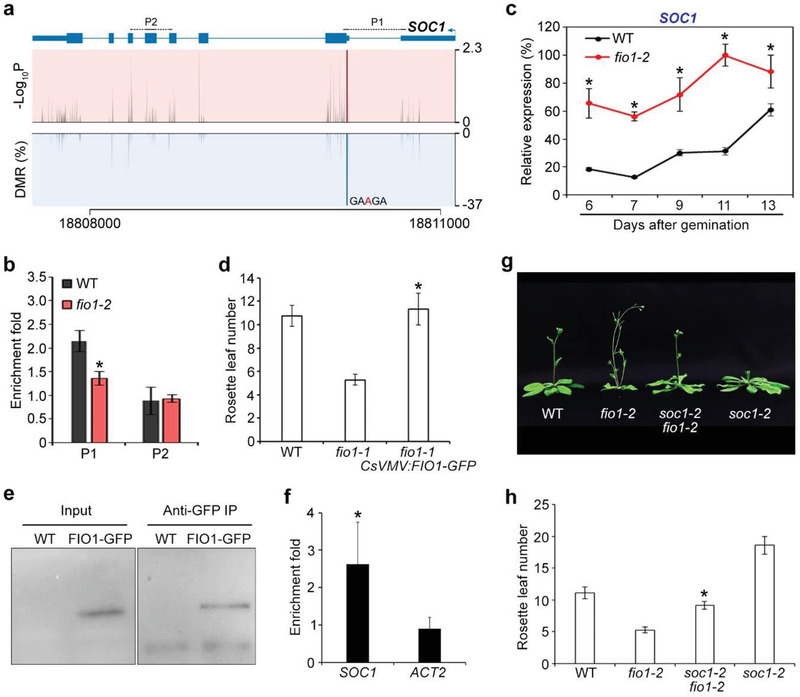
FIO1‐mediated m^6^A methylation modulates *SOC1* expression in flowering time control. a) Diagram showing the DMR, the corresponding *P* value, and the transcript sequence with an identified m^6^A site in *SOC1* transcripts. The gene structure is shown above. Thick and thin boxes represent exons and UTRs, respectively, and lines represent introns. The sequences amplified by the primers are labeled above the gene structure. b) Verification of the nanopore direct RNA sequencing result for *SOC1*. m^6^A‐IP‐qPCR was performed with 6‐day‐old wild‐type and *fio1‐2* seedlings. Error bars, mean ± SD; *n* = 3 biological replicates. Asterisk indicates a significant difference in m^6^A enrichment levels between *fio1‐2* and wild‐type seedlings (two‐tailed paired Student's *t*‐test, *P* < 0.05). c) Temporal expression pattern of *SOC1* in developing wild‐type and *fio1‐2* seedlings. Wild‐type and *fio1‐2* seedlings grown under long days were harvested for expression analysis. The expression levels were normalized to *TUB2* expression and then normalized to the highest expression level set as 100%. Error bars, mean ± SD; *n* = 3 biological replicates. Asterisks indicate significant differences between *fio1‐2* and wild‐type seedlings (two‐tailed paired Student's *t*‐test, *P* < 0.05). d) An *fio1‐1 CsVMV:FIO1‐GFP* transgenic line shows comparable flowering time to a wild‐type plant under long days. Error bars, mean ± SD; *n* = 15. Asterisk indicates a significant difference in the flowering time between *fio1‐1 CsVMV:FIO1‐GFP* and *fio1‐1* (two‐tailed paired Student's *t*‐test, *P* < 0.05). e) FIO1‐GFP can be detected and immunoprecipitated by anti‐GFP antibodies. Six‐day‐old wild‐type and *CsVMV:FIO1‐GFP* seedlings were harvested for analysis. Western blot was performed with the input and immunoprecipitated (IP) samples using anti‐GFP antibody. f) RNA immunoprecipitation assay reveals the direct binding of FIO1‐GFP to *SOC1* transcripts. Six‐day‐old wild‐type and *fio1‐1 CsVMV:FIO1‐GFP* seedlings grown under long days were harvested for RNA immunoprecipitation assay. Enrichment of *ACTIN2* (*ACT2*) was included as a negative control. Error bars, mean ± SD; *n* = 3 biological replicates. Asterisk indicates a significant difference in FIO1‐GFP enrichment on *SOC1* compared with the *ACT2* negative control (two‐tailed paired Student's *t*‐test, *P* < 0.05). g) A *soc1‐2 fio1‐2* double mutant flowers later than *fio1‐2*. h) Flowering time of *soc1‐2 fio1‐2* under long days. Error bars, mean ± SD; *n* = 15 plants. Asterisk indicates a significant difference in the flowering time between *soc1‐2 fio1‐2* and *fio1‐2* (two‐tailed paired Student's *t*‐test, *P* < 0.05).

In line with the early‐flowering phenotype observed in *fio1‐2* mutants (Figure 1d,e), the hypomethylated genes include a key flowering integrator *SOC1* ^[^ ^26^, ^27^ ^]^ (Figure S12 and Table S2, Supporting Information), which is among the differentially expressed genes in *fio1‐2* (Table S3, Supporting Information). *SOC1* transcript contains a hypomethylated site (GAm ^6^ AGA) in the 5′ UTR immediately upstream of the start codon (**Figure** **4**a). The reduced m^6^A levels on *SOC1* transcripts were further verified by m^6^A‐IP‐qPCR in *fio1‐2* mutants (Figure 4b). Consistent with the nanopore sequencing results, quantitative real‐time PCR revealed that *SOC1* expression was significantly upregulated in developing *fio1‐2* seedlings compared to wild‐type plants (Figure 4c). The reduction of m^6^A levels on *SOC1* transcripts and increased *SOC1* expression in *fio1‐2* were restored in *fio1‐2 gFIO1*, but not in *fio1‐2 gmFIO1* (Figure S13, Supporting Information). Moreover, FIO1‐GFP directly bound to *SOC1* transcripts in vivo as revealed by RNA immunoprecipitation followed by quantitative real‐time PCR assays with a functional *fio1‐1 CsVMV:FIO1‐GFP* line^[^ ^17^ ^]^ (Figure 4d–f), suggesting that FIO1 directly modulates m^6^A methylation on *SOC1* transcripts and its expression levels.

**Figure 5:**
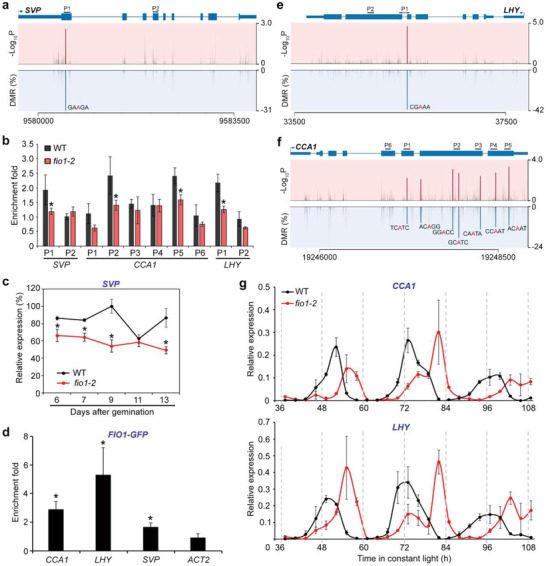
FIO1‐mediated m^6^A methylation modulates the expression pattern of genes acting upstream of *SOC1*. a) Schematic diagrams showing the DMRs, the corresponding *P* value, and the transcript sequence with an identified m^6^A site in *SVP* transcripts. The gene structure is shown above. Thick and thin boxes represent exons and UTRs, respectively, and lines represent introns. The sequences amplified by the primers are labeled above the gene structure. b) Verification of the nanopore direct RNA sequencing results for several genes acting upstream of *SOC1*. m^6^A‐IP‐qPCR was performed with 6‐day‐old wild‐type and *fio1‐2* seedlings. Error bars, mean ± SD; *n* = 3 biological replicates. Asterisks indicate significant differences in m^6^A enrichment levels between *fio1‐2* and wild‐type seedlings (two‐tailed paired Student's *t*‐test, *P* < 0.05). c) Temporal expression pattern of *SVP* in developing wild‐type and *fio1‐2* seedlings. Wild‐type and *fio1‐2* seedlings grown under long days were harvested for expression analysis. The expression levels were normalized to *TUB2* expression and then normalized to the highest expression level set as 100%. Error bars, mean ± SD; *n* = 3 biological replicates. Asterisks indicate significant differences between *fio1‐2* and wild‐type seedlings (two‐tailed paired Student's *t*‐test, *P* < 0.05). d) RNA immunoprecipitation assay reveals the direct binding of FIO1‐GFP to the transcripts of *SVP*, *CCA1*, and *LHY*. Six‐day‐old wild‐type and *fio1‐1 CsVMV:FIO1‐GFP* seedlings grown under long days were harvested for RNA immunoprecipitation assay. Enrichment of *ACT2* was included as a negative control. Error bars, mean ± SD; *n* = 3 biological replicates. Asterisks indicate significant differences in FIO1‐GFP enrichment on *SVP*, *CCA1*, and *LHY* compared with *ACT2* (two‐tailed paired Student's *t*‐test, *P* < 0.05). Schematic diagrams showing the DMRs, corresponding *P* values, and the transcript sequences with the identified m^6^A sites in e) *CCA1* and f) *LHY* transcripts. g) Disruption of *fio1‐2* lengthens the cycling periods of *CCA1* (upper panel) and *LHY* (lower panel). Wild‐type and *fio1‐2* seedlings were first entrained with 12 h light/12 h dark photoperiods for 9 days before being shifted to the constant light conditions at ZT 0. The samples were collected at 3 h interval from ZT 37 for 3 days. Expression levels of *CCA1* and *LHY* were determined by quantitative real‐time PCR and normalized to the expression of *TUB2*.

Interestingly, we also identified *SHORT VEGETATIVE PHASE* (*SVP*), a direct repressor of *SOC1*,^[^ ^26^ ^]^ was a hypomethylated gene in *fio1‐2* (**Figure** **5**a; Figure S12 and Table S2, Supporting Information). The reduced m^6^A level on *SVP* transcript was further confirmed by m^6^A‐IP‐qPCR (Figure 5b). *SVP* expression was significantly downregulated in developing *fio1‐2* seedlings compared to wild‐type plants (Figure 5c), which is in agreement with the early‐flowering phenotype of *fio1‐2*. FIO1‐GFP was associated with *SVP* transcripts (Figure 5d), indicating that FIO1 directly methylates *SVP* to regulate its expression. Indeed, the reduction of m^6^A levels on *SVP* transcripts and decreased *SVP* expression in *fio1‐2* were restored in *fio1‐2 gFIO1*, but not in *fio1‐2 gmFIO1* (Figure S13, Supporting Information).

Moreover, the central circadian oscillator genes *CIRCADIAN CLOCK ASSOCIATED 1* (*CCA1*) and *LATE ELONGATED HYPOCOTYL* (*LHY*)^[^ ^28^ ^]^ were also identified as hypomethylated genes in *fio1‐2* (Figure S12 and Table S2, Supporting Information). Interestingly, *CCA1* contains seven hypomethylated sites in its CDS involving different k‐mers, whereas *LHY* contains one hypomethylated sites (Figure 5e,f). Among them, several sites were verified with m^6^A‐IP‐qPCR (Figure 5b), implying that nanopore direct sequencing may identify modification sites below the detection threshold of antibody‐based approaches as similarly shown in mammalian cells.^[^ ^8^ ^]^ As *CCA1* and *LHY* are clock genes, we further examined the effect of FIO1 on their expression pattern, and found that disruption of *FIO1* lengthened the period length of the expression of *CCA1* and *LHY* (Figure 5g). This is consistent with the previous study showing that FIO1 controls period length in the circadian clock.^[^ ^17^ ^]^ FIO1‐GFP was associated with the *CCA1* and *LHY* transcripts (Figure 5d), indicating that FIO1 directly methylates *CCA1* and *LHY* mRNAs. As a major output of the circadian oscillation is its effect on flowering time, we consequently observed the upregulation of two circadian‐regulated genes, *CONSTANS* (*CO*)^[^ ^29^ ^]^ and its immediate downstream gene *FLOWERING LOCUS T* (*FT*),^[^ ^30^, ^31^ ^]^ in *fio1‐2* mutants, although *CO* and *FT* were not the methylated targets of FIO1 (Figure S14a–c and Table S2, Supporting Information). As FT positively regulates *SOC1* expression,^[^ ^32^ ^]^ FIO1 effect on circadian clock genes indirectly contributes to repression of *SOC1*. Taken together, these observations indicate that FIO1‐mediated m^6^A methylation suppresses *SOC1* expression through both directly downregulating *SOC1* transcript abundance and indirectly affecting the mRNA expression of its upstream regulators, including *SVP*, *CO*, and *FT*. Indeed, *soc1‐2* greatly suppressed the early‐flowering phenotype of *fio1‐2* (Figure 4g,h), further supporting that FIO1 functions through *SOC1* to prevent premature flowering.

**Figure 6:**
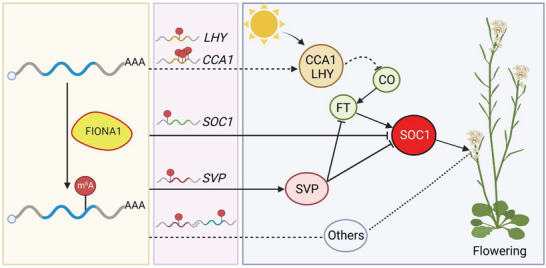
A proposed model depicting the function of FIO1 in m^6^A modification and flowering time control in *Arabidopsis*. FIO1 is involved in depositing m^6^A methylation mainly on the CDS of a subset of protein‐coding transcripts. FIO1‐mediated m^6^A methylation determines the expression of the central flowering integrator *SOC1* through both directly downregulating *SOC1* transcript abundance and indirectly affecting the mRNA expression of its upstream regulators, including *SVP*, *CO*, and *FT*, to prevent premature flowering. FIO1 directly methylates the transcripts of *SOC1*, *SVP*, *CCA1*, *LHY*, and possibly other genes in regulating the floral transition. FIO1 modulates the transcript levels of *SVP*, a direct repressor of *SOC1*. FIO1 is also required for the normal cycling periods of *CCA1* and *LHY*, which in turn affect the expression of *CO* and *FT*, thus influencing *SOC1* expression. Direct stimulatory interactions are indicated by arrows, and direct or indirect inhibitory interactions are indicated by T‐bars or dashed lines with T‐bars, respectively. The dashed line with arrow indicates that FIO1 regulates the cycling periods of *CCA1* and *LHY*, while the dashed lines indicate possible effects of other unknown targets of FIO1 on flowering. Created with BioRender.com

In this study, we have revealed that FIO1 functions as an m^6^A methyltransferase and regulates flowering time in *Arabidopsis* (**Figure** **6**). Compared to m^6^A deposition mainly near the stop codon and 3′ UTR by other known m^6^A writers,^[^ ^9^, ^13^ ^]^ the hypomethylated sites in *fio1* mutants are mainly located in the CDS region, implying that FIO1 acts independently of other known m^6^A writers. Similarly, METTL16‐dependent m^6^A peaks mainly found in the introns and intron–exon boundaries in human genes are also distinct from those m^6^A sites found in 3′ UTRs.^[^ ^20^ ^]^ FIO1 does not regulate the expression levels of known m^6^A writer genes or is not involved in the multicomponent m^6^A writer complex (Figure S6b, Supporting Information). Moreover, the consensus motif YHm ^6^ AGA mostly enriched in the FIO1‐methylated targets is different from the motif RRm ^6^ ACH associated with the other known m^6^A writers.^[^ ^9^, ^13^ ^]^ Thus, different m^6^A methyltransferases exist and function concurrently to establish the m^6^A landscape in plants.

Unlike other known methyltransferases in *Arabidopsis*, FIO1‐mediated m^6^A modification uniquely regulates the key developmental transition from vegetative to reproductive growth through at least directly and indirectly downregulating the mRNA abundance of a central flowering integrator *SOC1* (Figure 6). Interestingly, in addition to the FIO1‐dependent m^6^A sites in their 5′UTR/CDS junction and CDS, both *SOC1* and *SVP* mRNAs extracted from various developmental stages also bear FIP37‐dependent m^6^A peaks or VIR‐dependent m^6^A sites in their 3′ UTRs (Figure S15a, Supporting Information). However, unlike FIO1, the other known m^6^A writers, including FIP37 and VIR, did not significantly affect the expression of *SOC1* and *SVP* before the floral transition (Figure S15b, Supporting Information). These observations imply that although FIO1 and the other known m^6^A writers could methylate the same mRNAs, different methylation sites could result in different cellular fates of these mRNAs. Moreover, as the hypomethylated genes in *fio1‐2* mutants also include other clock component genes or chromatin regulatory genes (Figure S12 and Table S2, Supporting Information), it would be interesting to further investigate whether FIO1 effect on these genes may also contribute to modulation of flowering time under different environmental and developmental conditions.

METTL16, the ortholog of FIO1 in human, also functions as a U6 snRNA m^6^A methyltransferase.^[^ ^20^ ^]^ In our study, we found that m^6^A level in U6 snRNAs were indeed reduced in *fio1‐2* (Figure S16, Supporting Information), suggesting the conserved function of FIO1 and METTL16 in methylating U6 snRNAs. In addition, we also observed that *MAT1/2/3/4*,^[^ ^33^, ^34^, ^35^ ^]^ all four *Arabidopsis* orthologous genes of the human SAM synthetase gene *MTA2A*, contain hypomethylated sites in *fio1* mutants (Figures S17 and S18a, Supporting Information), implying a scenario similar to methylating *MTA2A* by METTL16 in human. However, the *MAT* genes in *Arabidopsis* do not possess the hairpin structure in the 3′ UTR, which is present in human *MTA2A* and is m^6^A methylated by METTL16,^[^ ^20^ ^]^ indicating that FIO1 and METTL16 may methylate on their targets in different manners. Moreover, the expression levels of these *MAT* genes were not altered in *fio1* mutants (Figure S18b, Supporting Information), raising the possibility that FIO1 may affect *MAT* transcript processing in other aspects, such as translocation or translation.

## 3 Conclusion

Overall, our study elucidates the function of FIO1 as a unique m^6^A methyltransferase independently of other known m^6^A methyltransferases in establishing appropriate levels of m^6^A modifications preferentially in the coding sequences of a subset of protein coding transcripts. FIO1‐mediated m^6^A methylation is associated with transcript abundance and alternative polyadenylation, but has a limited effect on alternative splicing. We further reveal that FIO1‐mediated m^6^A methylation determines the mRNA abundance of a central flowering integrator *SOC1* and its upstream regulators, thus preventing early flowering. Our findings provide novel insights into understanding of m^6^A landscape, the associated new players and their biological functions in plants, and also shed light into the cognate mechanisms of METTL16‐mediated RNA modification in animals.

## 4 Experimental Section

### Plant Materials and Growth Conditions

Seeds of *Arabidopsis* (*A. thaliana*) were placed on soil or Murashige and Skoog (MS) medium and stratified at 4 °C in darkness for 3 days before they were grown under long days (16 h light/8 h dark) or short days (8 h light/16 h dark) at 23 ± 2 °C. The seeds of *fio1‐2* (SALK_209355) were ordered from the *Arabidopsis* Information Resource, and the seeds of *fio1‐1* mutants and *fio1‐1 CsVMV:FIO1‐GFP* transgenic plants were kindly provided by Prof. Hong Gil Nam (Daegu Gyeongbuk Institute of Science and Technology). *Agrobacterium tumefaciens*‐mediated transformation of *Arabidopsis* mutant plants was performed using the floral dipping method.^[^ ^36^ ^]^

### Plasmid Construction

To construct *gFIO1*, the 4.7 kb *FIO1* genomic sequence including 2.0 kb upstream sequence, 2.7 kb full‐length coding region plus intron, and the 3′ UTR was amplified and ligated into a pENTR vector. The *gFIO1* construct also served as a template for generating *gmFIO1*, in which the key catalytic residues “NPPF” were mutated to “NAAF.” Both *gFIO1* and *gmFIO1* vectors were introduced to the destination vector through the Gateway LR recombination assay (Invitrogen).

### Dot Blot Analysis

Dot blot analysis was performed as previously described.^[^ ^13^ ^]^ Briefly, denatured mRNA was spotted onto a Hybond‐N+ membrane (Amersham) that is optimized for nucleic acid transfer. The membrane was UV crosslinked in a Stratalinker 2400 UV Crosslinked (Stratagene), before it was washed by 1× PBST buffer for 5 min at room temperature. The membrane was then blocked with 5% of nonfat milk in PBST, and incubated with anti‐m^6^A antibody (1:250, Synaptic Systems) overnight at 4 °C. After incubating with horseradish‐peroxidase‐conjugated anti‐rabbit IgG secondary antibody (Santa Cruz), the membrane was visualized with an ECL Western Blotting Detecting Kit (Thermo) in a ChemiDoc Touch Imaging System (Bio‐rad).

### Measurement of m^6^A/A Ratio by LC‐MS/MS Analysis

Total RNA was extracted with the RNeasy Plus Mini Kit and mRNA was purified from the total RNA using Dynabeads Oligo(dT)_25_ (Invitrogen). mRNA was digested into single ribonucleosides as previously described,^[^ ^37^ ^]^ followed by clean‐up with chloroform. Samples were subjected to LC‐MS/MS analysis on a SCIEX QTRAP 6500 spectrometer. Multiple reaction monitoring mode was used to detect A and m^6^A with mass transitions at 268.0 to 136.0 and 282.0 to 150.1, respectively.

### Nanopore Direct RNA Sequencing

Six‐day‐old seedlings of wild‐type and *fio1‐2* were harvested, and total RNA was extracted with the TRIzol reagent (Invitrogen). mRNA was then purified from the total RNA using Dynabeads Oligo(dT)_25_ (Invitrogen). The quantity and quality of mRNA were determined with an Agilent Bioanalyzer system. Each library was prepared with around 750 ng of mRNA using the Nanopore direct RNA sequencing kit (SQK‐RNA002, Oxford Nanopore Technologies). The prepared libraries were loaded onto FLO‐MIN106 flow cells and sequenced with the GridION device. The run duration for each library was ≈40‐72 h. The raw fast5 data were base called with Guppy 4.2.3 with the high accuracy mode to generate FASTQ files.

### m^6^A Modification Site Analysis

The FASTQ reads were mapped to the reference transcriptome of TAIR10 by Minimap2 2.20.^[^ ^23^ ^]^ Alignment was subsequently converted to BAM file by Samtools^[^ ^38^ ^]^ and Nanopolish Eventalign v0.13^[^ ^24^ ^]^ was used for signal segmentation. Both aligned reads and events with the reference annotation (Ensemble plant release 50) were processed with Xpore^[^ ^8^ ^]^ to detect differential m^6^A modification site.

### Differential Expression Analysis with Nanopore Reads

Reads count for wild‐type and *fio1‐2* was performed with Subread v2.0.3^[^ ^39^ ^]^ and featureCounts v2.0.2^[^ ^40^ ^]^ in the long‐read mode. Differentially expressed genes were identified with DESeq2 v1.32.^[^ ^41^ ^]^

### Poly(A) Length Estimation

Nanopolish v0.13^[^ ^24^ ^]^ was performed to analyze the poly(A) signal from the fast5 file. Differences between *fio1‐2* and wild‐type were identified using a Mann–Whitney test.

### Alternative Splicing Analysis

Full‐length alternative isoform analysis of RNA (FLAIR) v1.5^[^ ^42^ ^]^ was performed to detect the differential isoform usage and differential alternative splicing events between wild‐type and *fio1‐2* with the nanopore reads following the standard workflow.

### Identification of Alternative 3′ End Positions

3′ end usage of wild‐type and *fio1‐2* plants was recorded and reads that overlapped with each gene locus less than 20% were filtered. To identify the differential usage of each position between wild‐type and *fio1‐2*, the Kolmogorov–Smirnov test was performed followed by multiple testing correction with the Benjamini–Hochberg method. The differential 3′ end usage results were filtered for an FDR of <0.05. Subsequently, the 3′ end usage to total number of reads of each gene locus was normalized, and the difference between normalized numbers of *fio1‐2* and wild‐type of each site was used to get the minimum and maximum numbers, which represent the most reduced and increased 3′ end usage sites, respectively. The difference between most reduced and most increased 3′ end usage sites was used to estimate the direction and distance of each change.

### In Vitro Methylation Assay

The in vitro methylation assay was performed as previously described.^[^ ^43^, ^44^ ^]^ An RNA probe (GCCAGAGCCAGAGCCAGAGCCAGA) containing four repeats of the consensus m^6^A motif recognized by FIO1 was synthesized. The full‐length coding sequence of *FIO1* was cloned into pGEX‐6p‐2 vector (GE Healthcare). This construct further served as a template for generating mFIO1, in which the key catalytic residues “NPPF” were mutated to “NAAF.” GST, GST‐FIO1, and GST‐mFIO1 proteins were expressed in *Escherichia coli* Rosetta (DE3) cells by induction with isopropyl *β*‐D‐1‐thiogalactopyranoside (IPTG) at 16 °C overnight, and purified with Glutathione Sepharose (Amersham Bioscience).

### Expression Analysis

Total RNA was extracted with the RNeasy Plus Mini Kit (QIAGEN) and reversed transcribed with the M‐MLV Reverse Transcriptase (Promega) following the manufacturers’ protocols. Quantitative real‐time PCR was performed on three biological replicates using 7900HT Fast Real‐Time PCR systems (Applied Biosystems) with PowerUp SYBR Green Master Mix (Applied Biosystems). *TUB2* expression was used as an internal control. The difference between the cycle threshold (Ct) of target genes and the Ct of control primers (∆Ct = Ct_target gene_ − Ct_control_) was used to calculate the normalized expression of target genes. The primers used for gene expression analysis are listed in Table S6 in the Supporting Information.

### m^6^A‐IP‐qPCR

m^6^A‐IP‐qPCR was performed as previously described.^[^ ^6^, ^13^ ^]^ Total RNA was extracted from wild‐type and *fio1‐2* seedlings with the RNeasy Plus Mini Kit (QIAGEN) and fragmented into ≈200‐nucleotide‐long fragments. Fragmented RNA was incubated with anti‐m^6^A antibody (202‐003; Synaptic Systems) in IP buffer (10 × 10^−3^ m Tris‐HCl pH 7.4, 150 × 10^−3^ m NaCl, 0.1% Igepal CA‐630) supplemented with RNasin Plus RNase inhibitor (Progema) for 2 h at 4 °C with gentle rotation. This mixture was subsequently incubated with Protein A/G Plus Agarose (Santa Cruz) that was prebound with BSA for an additional 2 h at 4 °C with gentle rotation. After extensive wash with IP buffer, the bound RNA was eluted from the beads with IP buffer plus 6.7 × 10^−3^ m *N* ^6^‐methyladenosine 5′‐monophosphate sodium salt (Sigma) and precipitated by ethanol. The input and immunoprecipitated RNA were then reverse transcribed using random hexamers (Invitrogen) with M‐MLV Reverse Transcriptase (Promega). Relative enrichment of each fragment was determined by quantitative real‐time PCR and calculated as previously described.^[^ ^13^ ^]^ A *TUB2* fragment was used as internal control. The primers used for m^6^A‐IP‐qPCR analysis are listed in Table S6 in the Supporting information.

### RNA Immunoprecipitation

RNA immunoprecipitation was performed as previously published^[^ ^45^ ^]^ with minor modifications. Two grams of 6‐day‐old seedlings of *FIO1‐GFP* were collected and fixed with 1% formaldehyde under vacuum for 20 min. The fixed tissues were homogenized and lysed with cell lysis buffer (50 × 10^−3^ m Tris‐HCl, pH 7.5, 150 × 10^−3^ m NaCl, 4 × 10^−3^ m MgCl_2_, 0.25% Igepal CA‐630, 1% SDS, 0.25% sodium deoxycholate, and 5 × 10^−3^ m DTT) supplemented with Complete EDTA‐free Protease Inhibitor Cocktail (Roche) and RNasin Plus RNase inhibitor (Promega). The protein extract was subjected to immunoprecipitation with anti‐GFP antibody (Invitrogen) bound to Protein A/G Plus Agarose (Santa Cruz). After incubating at 4 °C for 4 h, the beads were washed extensively with the washing buffer (50 × 10^−3^ m Tris‐HCl, pH 7.5, 500 × 10^−3^ m NaCl, 4 × 10^−3^ m MgCl_2_, 0.5% Igepal CA‐630, 1% SDS, 0.5% sodium deoxycholate, 2 m urea, and 2 × 10^−3^ m DTT) supplemented with RNasin Plus RNase inhibitor for four times. The beads were subsequently treated with Turbo DNase (Invitrogen) at 37 °C for 10 min. RNA was extracted from the input and beads with RNeasy Plus Mini Kit (QIAGEN) and reversed transcribed with random hexamers (Invitrogen) using the M‐MLV Reverse Transcriptase (Promega) following the manufacturers’ protocols. Relative enrichment of each gene was determined by quantitative real‐time PCR and calculated as previously described.^[^ ^13^ ^]^ The primers used for RNA immunoprecipitation analysis are listed in Table S6 in the Supporting information.

### Yeast Two‐Hybrid

To construct AD‐FIO1, the coding sequence of *FIO1* was amplified and ligated into pGADT7 (Clontech). The coding sequences of MTA, MTB, HAKAI, and FIP37 were amplified and ligated into pGBKT7 (Clontech). The yeast two‐hybrid assay was performed with the yeast strain AH109 using the Yeastmaker Yeast Transformation System according to the manufacturer's instructions (Clontech).

### Data Availability

The nanopore direct RNA sequencing data described in this study have been deposited in NCBI Sequence Read Archive (SRA) database with the accession number: PRJNA749003. All the other data are available from the corresponding authors upon request.

### Statistical Analysis

Statistical details of the experiments are available in figure legends, including the statistical test used and exact value of *n*. The significance of the data between experimental groups was determined by two sided Mann–Whitney test, Kolmogorov–Smirnov test, Benjamini–Hochberg method, or two‐tailed paired Student's *t*‐test. A *P* value less than 0.05 represented a statistically significant difference, unless otherwise stated. *P* values of differential modification rates were determined by Xpore from *z*‐test of the differential modification rates.

## Conflict of Interest

The authors declare no conflict of interest.

## Author Contributions

T.X., H.Y., and L.S. conceived and designed this study. T.X., X.W., C.E.W., F.S., Y.Z., Z.L., and L.S. performed the experiments. T.X., S.Z., H.Y., and L.S. analyzed data. T.X., H.Y., and L.S. wrote the paper. All authors read and approved the manuscript.

## Supporting information

Supporting Information

Supplemental Table S1‐S6

## Data Availability

The data that support the findings of this study are openly available in NCBI Sequence Read Archive (SRA) database at https://www.ncbi.nlm.nih.gov/sra/PRJNA749003, reference number 749003.
