# Supplementary material for: FIONA1‐Mediated m6A Modification Regulates the Floral Transition in Arabidopsis
Source: Adv Sci (Weinh). 2022 Jan 5;9(6):2103628. doi: 10.1002/advs.202103628 (PMC8867147; doi:10.1002/advs.202103628)
Supplement: Supplementary file 1 — Supporting Information [file ADVS-9-2103628-s002.pdf]

ADVANCED SCIENCE
Open Access

Supporting Information

for *Adv. Sci.*, DOI: 10.1002/advs.202103628

FIONA1-Mediated m^{6}A Modification Regulates the Floral Transition in *Arabidopsis*

Tao Xu, Xiaowei Wu, Chui Eng Wong, Sheng Fan, Yu Zhang, Songyao Zhang, Zhe Liang, Hao Yu, and Lisha Shen**

Supporting Information

FIONA1-Mediated m^{6}A Modification Regulates the Floral Transition in *Arabidopsis*

Tao Xu, Xiaowei Wu, Chui Eng Wong, Sheng Fan, Yu Zhang, Songyao Zhang, Zhe Liang, Hao Yu, and Lisha Shen**

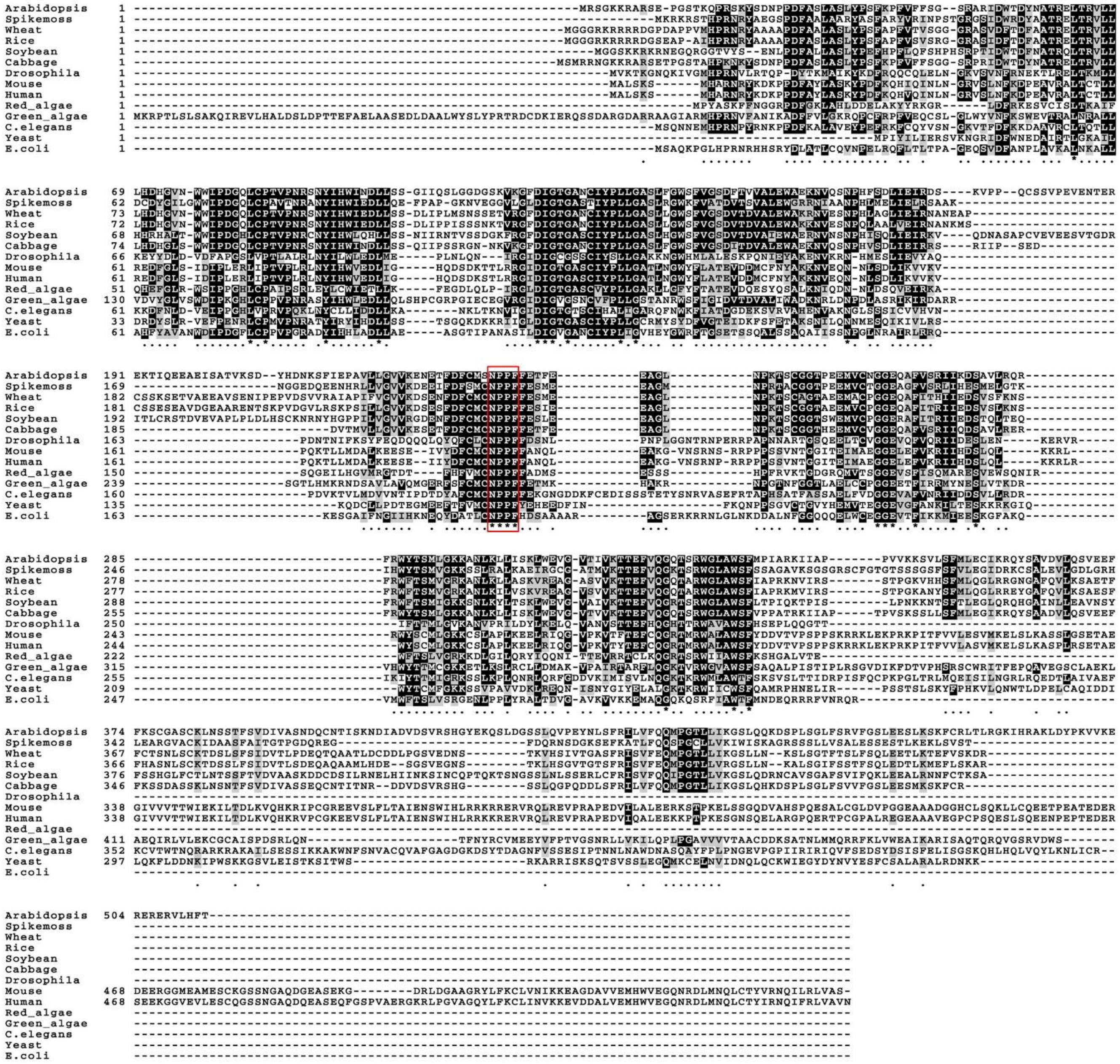

Figure S1. Sequence alignment of FIO1 and its homologs in other organisms. Conserved residues are shown in black, while similar residues are shown in grey. The highly conserved key catalytic residues NPPF were labeled with a red box. Amino acid sequences of *Arabidopsis* FIO1 (F4IGH3_ARATH; *Arabidopsis thaliana*) and its homologs from various organisms, including Spikemoss (D8S370_SELML, *Selaginella moellendorffii*), wheat (UPI0003D4495F, *Triticum aestivum*), rice (Q6YUR7_ORYSJ, *Oryza sativa* subsp. *japonica*), soybean (I1K375_SOYBN, *Glycine max*), Cabbage (M4ENF3_BRARP, *Brassica rapa* subsp. *Pekinensis*), Drosophila (MET16_DROME, *Drosophila melanogaster*), mouse (MET16_MOUSE, *Mus musculus*), human (MET16_HUMAN, *Homo sapiens*), Red algae (M2Y9P9_GALSU, *Galdieria sulphuraria*), green algae (C1FD75_MICCC, *Micromonas commode*), C. elegans (Q09357_CAEEL, *Caenorhabditis elegans*), yeast (O42662_SCHPO, *Schizosaccharomyces pombe*), and E. coli (RLMF_ECOLI, *Escherichia coli*), are obtained from UniProt and aligned.

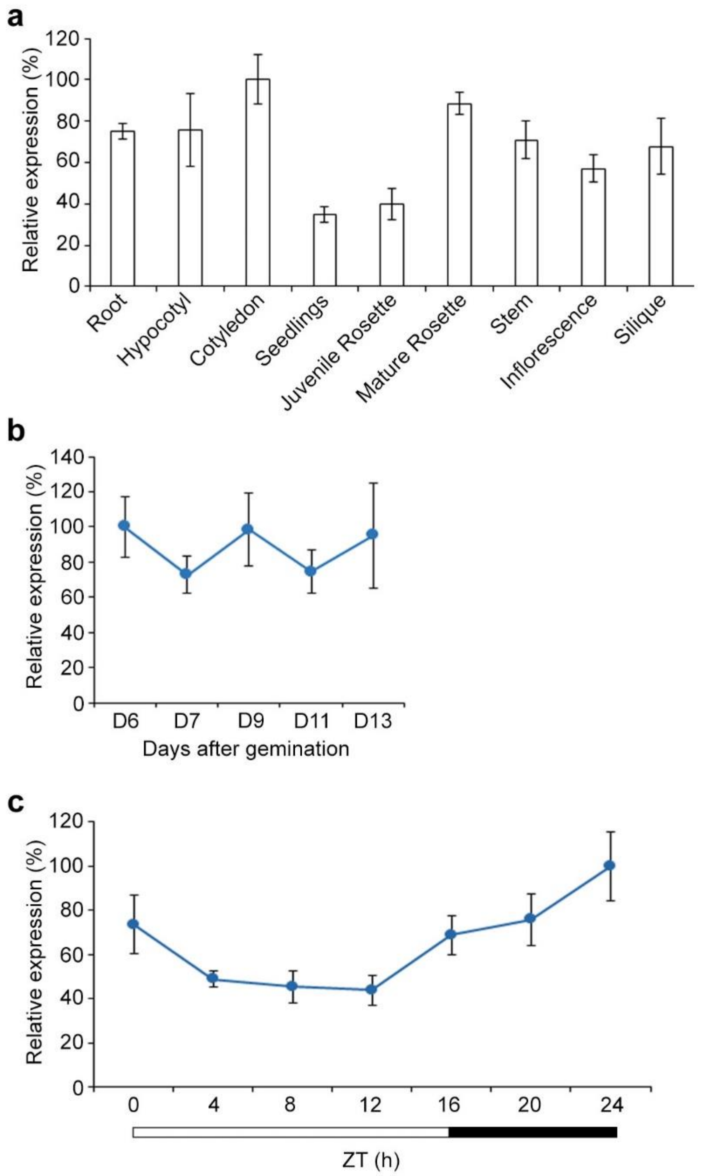

Figure S2. Expression pattern of *FIO1*. a) Expression pattern of *FIO1* in various *Arabidopsis* tissues. b) Temporal expression of *FIO1* during seedling development. Seedlings at different developmental stages grown under long days (LDs) were harvested for expression analysis. c) Expression of *FIO1* does not obviously oscillate within a 24-h cycle under LDs. Six-day-old seedlings grown under LDs were harvested at different time points expressed in hours as Zeitgeber time (ZT). *FIO1* expression (a-c) in wild-type Col plants was determined by quantitative real-time PCR analysis. Results were normalized against the expression levels of *TUB2* and the maximal expression level of *FIO1* in each panel was set as 100%. Error bars, mean ± SD; n = 3 biological replicates.

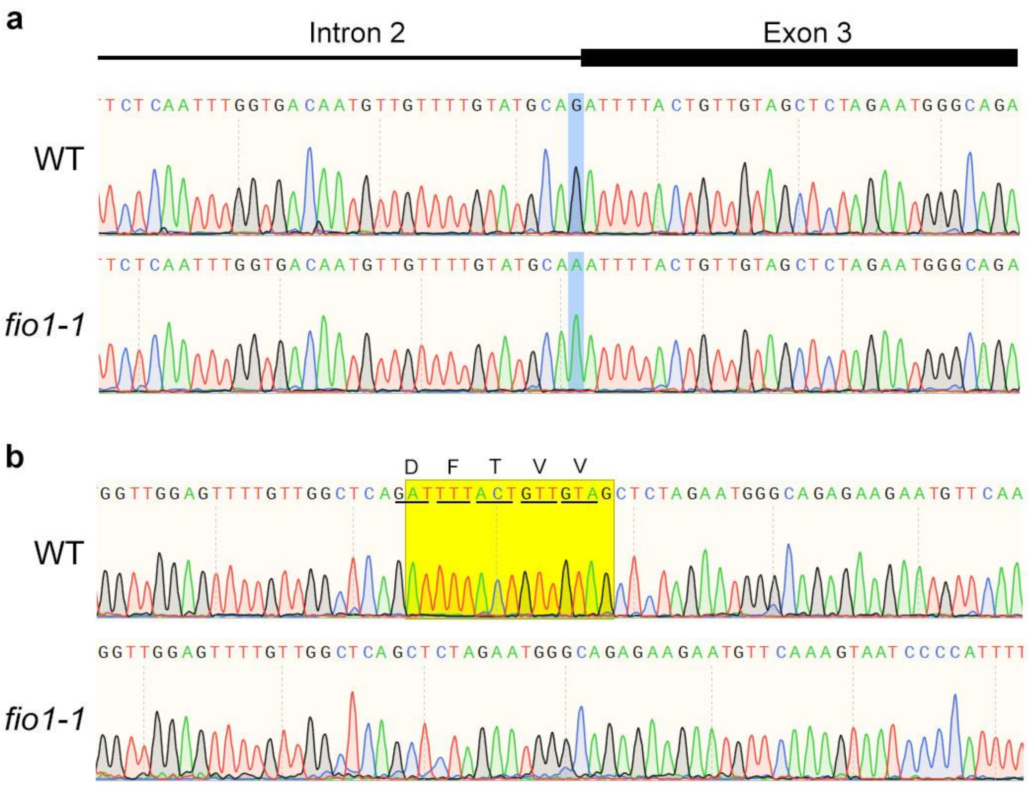

Figure S3. Sequencing of the *fio1-1* mutation site. a) Sequencing chromatographs showing the G to A (highlighted in blue) conversion in the splice acceptor site of the 2nd intron of the *FIO1* genomic sequence in *fio1-1*. b) Sequencing chromatographs showing a 15-bp deletion (highlighted in yellow) in the *fio1-1* cDNA sequence. This results in a 5 amino acid deletion (DFTVV) in the FIO1 protein sequence in *fio1-1*.

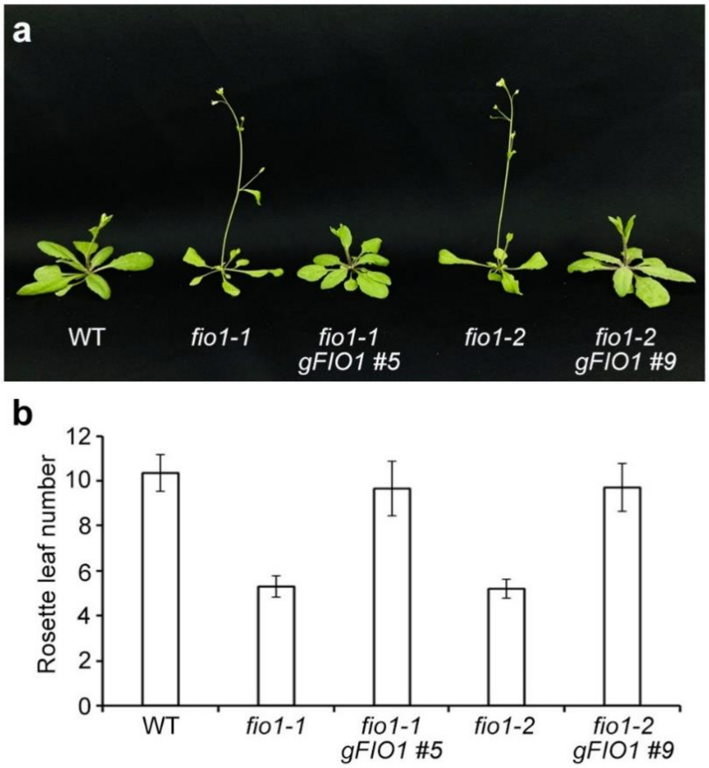

Figure S4. A genomic fragment of *FIO1* (*gFIO1*) fully complements the early-flowering phenotype of *fio1* mutants. a) Representative lines of *fio1-1 gFIO1* and *fio1-2 gFIO1* show comparable flowering time to a wild-type plant under LDs. b) Flowering time of representative lines of *fio1-1 gFIO1* and *fio1-2 gFIO1* under LDs. Error bars, mean ± SD; n = 20.

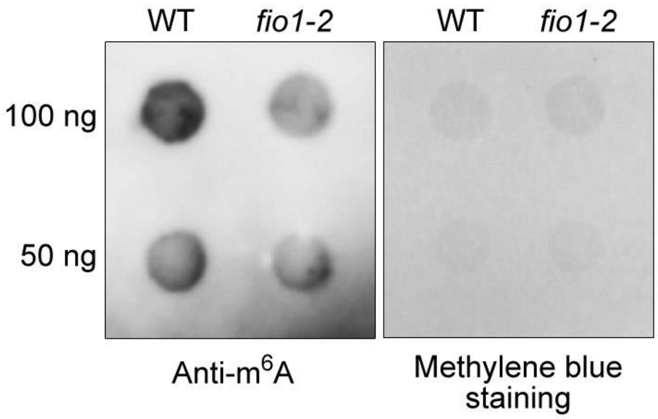

Figure S5. Dot blot analysis of m^6A levels in total RNA isolated from 6-day-old wild-type and *fio1-2* seedlings. Methylene blue staining of the membrane serves as a loading control.

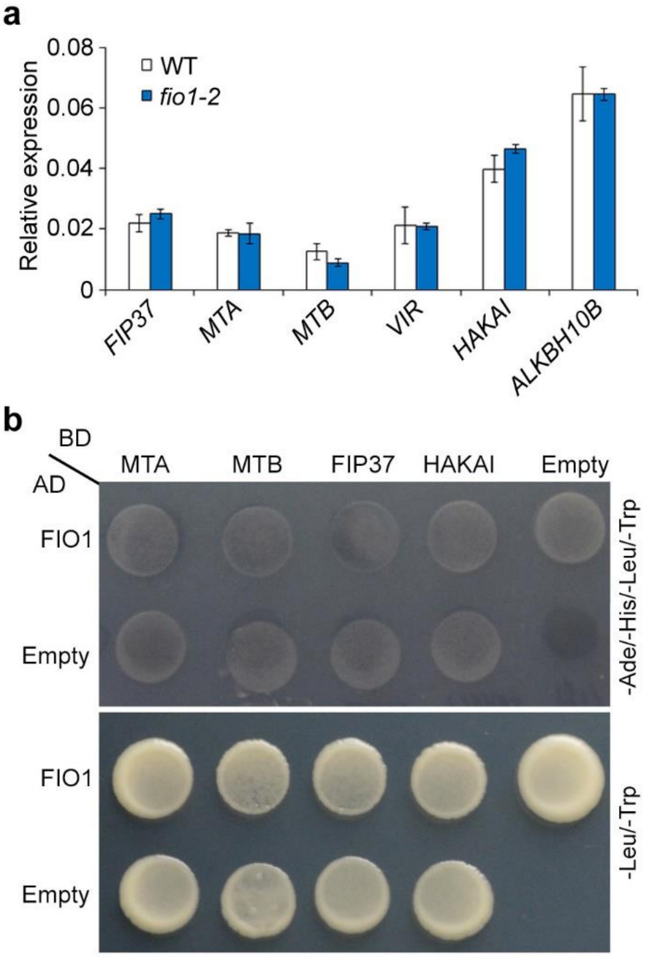

Figure S6. FIO1 may act independently of the other known m^{6}A writers. a) Expression of the known m^{6}A writer genes including *FIP37*, *MTA*, *MTB*, *VIR* and *HAKAI*, and the known m^{6}A eraser gene *ALKBH10B* in 6-day-old wild-type and *fio1-2* seedlings under LDs determined by real-time PCR. Error bars, mean ± SD; n = 3 biological replicates. b) Yeast two-hybrid indicates no direct interaction between FIO1 and known m^{6}A writer proteins. Transformed yeast cells were grown on SD-Ade/-His/-Leu/-Trp and SD-Leu/-Trp mediums.

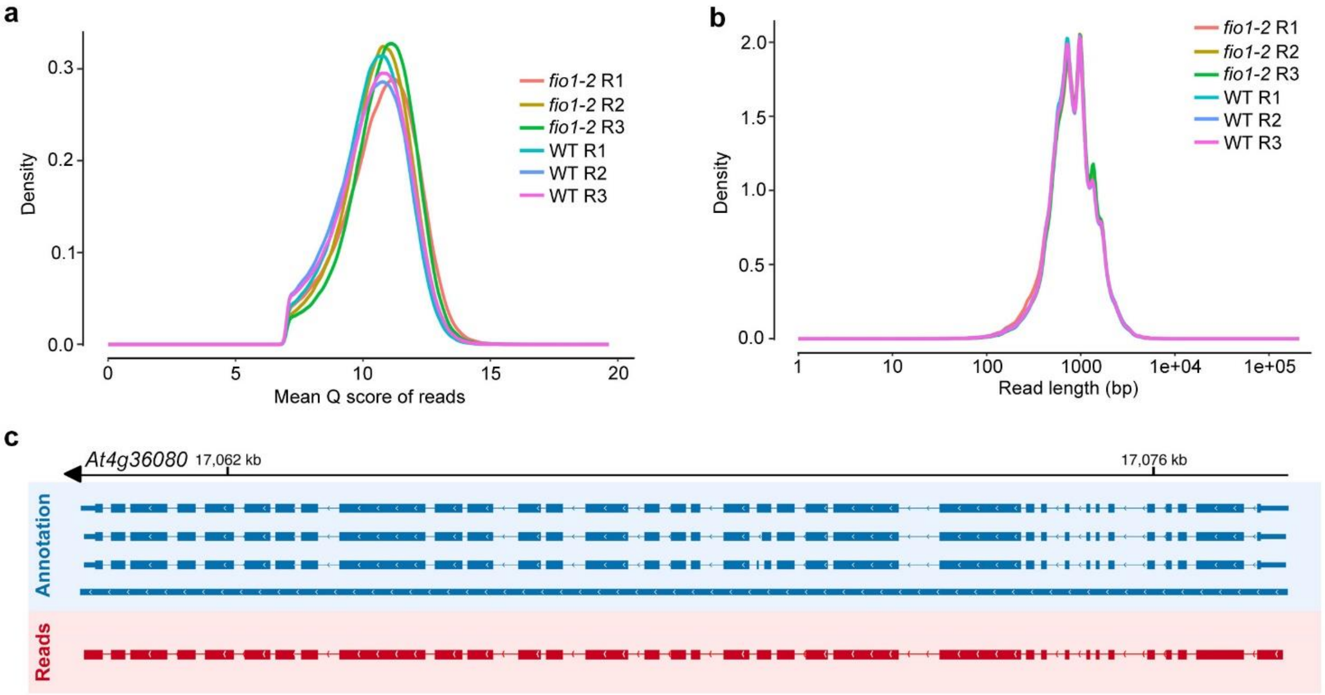

Figure S7. Analysis of nanopore reads. a) Distribution of Q scores of the nanopore reads. High-quality reads with Q score > 7 were used for further analysis. b) Distribution of continuous read length of the nanopore reads. c) An example of a long read of 11,624 nt from the *At4g36080* locus. The long nanopore reads (red color) was aligned well with the annotated locus (blue color) from TAIR (www.arabidopsis.org).

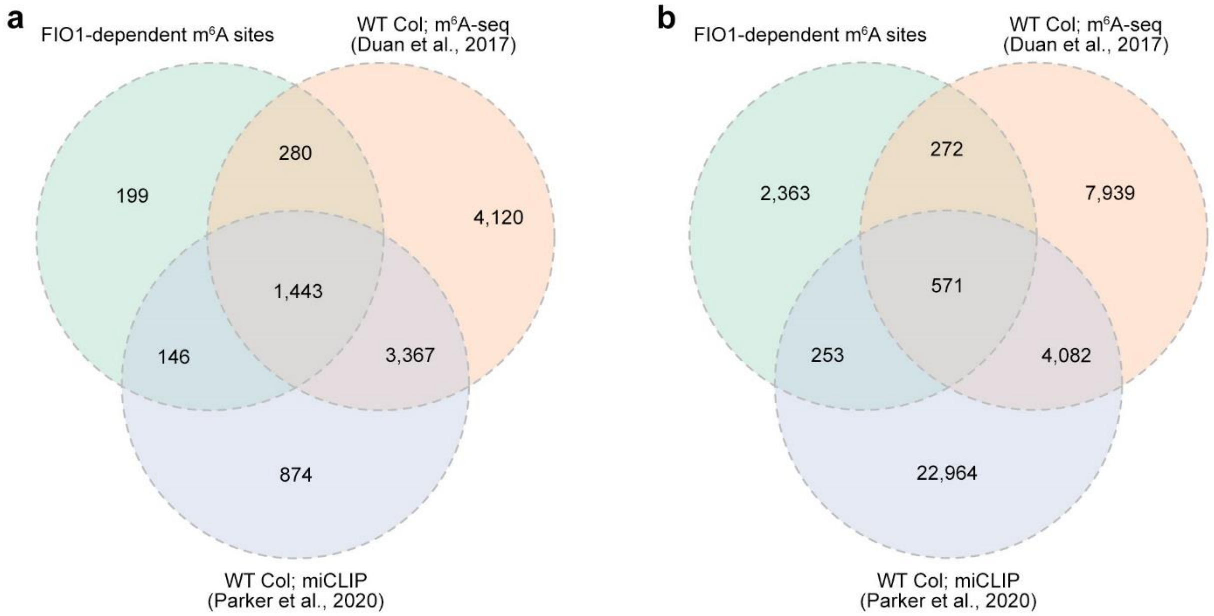

Figure S8. Comparison of FIO1-dependent m^6A sites with those m^6A sites revealed by m^6A-seq and miCLIP on the transcript basis (a) and the m^6A-site basis (b). The m^6A-seq[21] and miCLIP[9] data obtained from 2-week-old seedlings were used for this comparison.

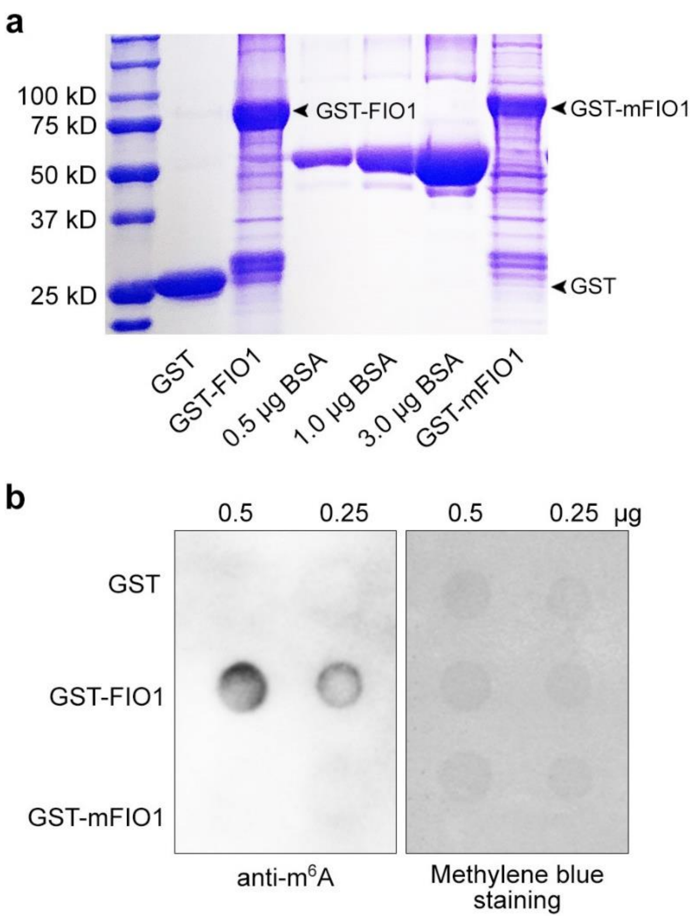

Figure S9. *In vitro* m^{6}A methylation assay. a) Expression of recombinant GST-FIO1 and GST-mFIO1 proteins. Arrowheads indicate the expression of GST, GST-FIO1 and GST-mFIO1 recombinant proteins. The key catalytic residues "NPPF" were mutated to "NAAF" in GST-mFIO1. Different amounts of BSA protein were included as controls. b) Examination of m^{6}A levels by dot blot analysis in RNA purified from the m^{6}A methylation assay. RNA oligo (GCCAGAGCCAGAGCCAGAGCCAGA) containing four repeats of the consensus m^{6}A motif recognized by FIO1 was incubated with GST, GST-FIO1 and GST-mFIO1, after which RNA was purified for examination of m^{6}A levels by dot blot analysis. Methylene blue staining of the membrane serves as a loading control.

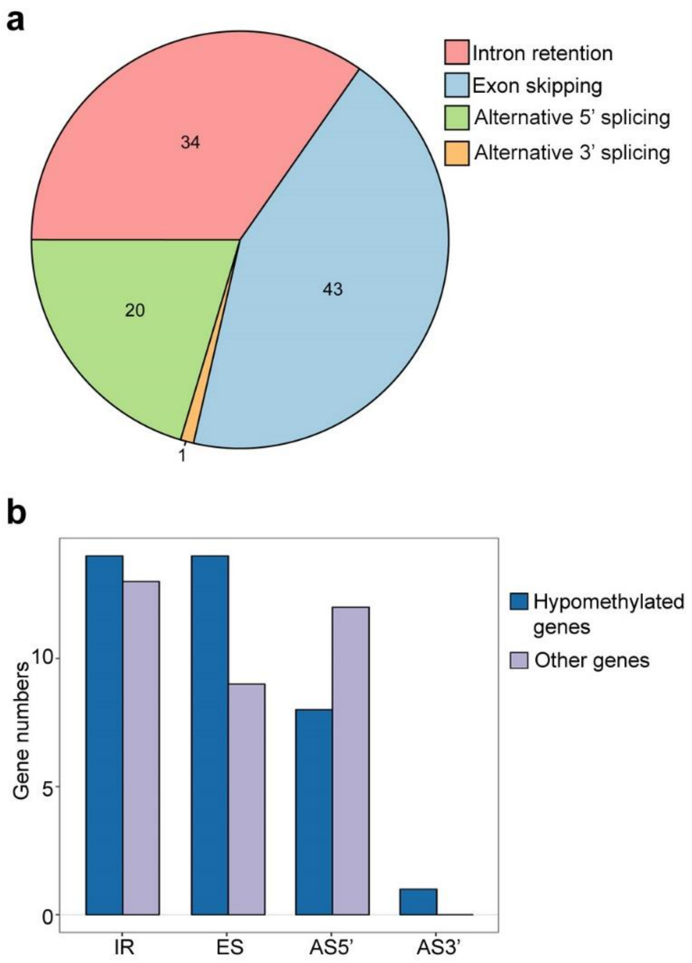

Figure S10. FIO1 does not greatly affect alternative splicing. a) Pie chart showing numbers of differential alternative splicing events in each category detected in *fio1-2* mutants. b) Comparison of numbers of hypomethylated genes and other genes with differential alternative splicing events. IR, intron retention; ES, exon skipping; AS5', alternative 5' splicing; AS3', alternative 3' splicing.

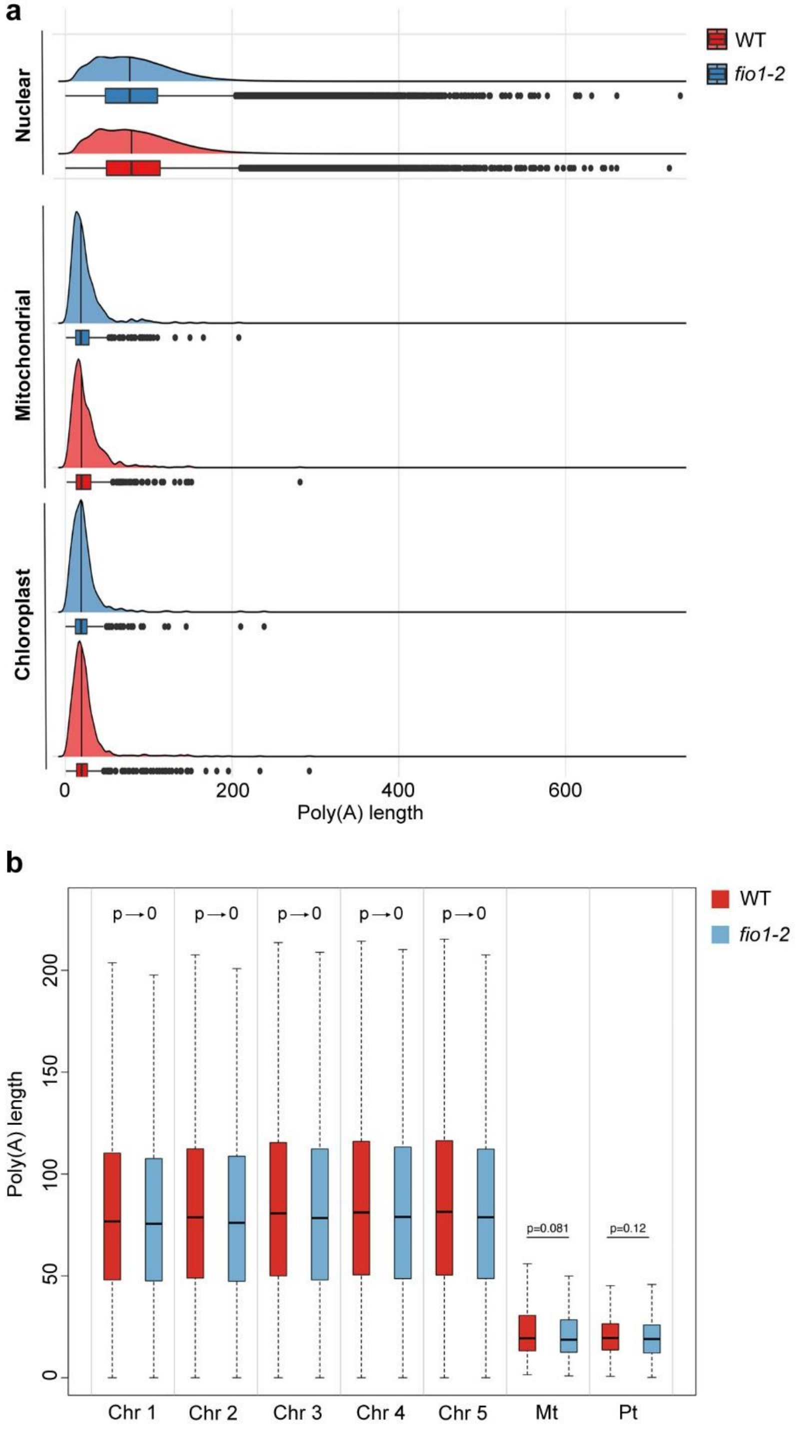

Figure S11. Analysis of poly(A) tail length in wild-type and *fio1-2* plants with nanopore reads. a) Distribution of poly(A) tail lengths of the nuclear-, mitochondrial- and chloroplast-encoded transcripts in wild-type and *fio1-2* plants. b) Box plot showing the poly(A) tail length of transcripts encoded by various chromosomes and mitochondrial and plastid genomes in wild-type and *fio1-2* plants. Chr, chromosome; Mt, mitochondrial; Pt, plastid.

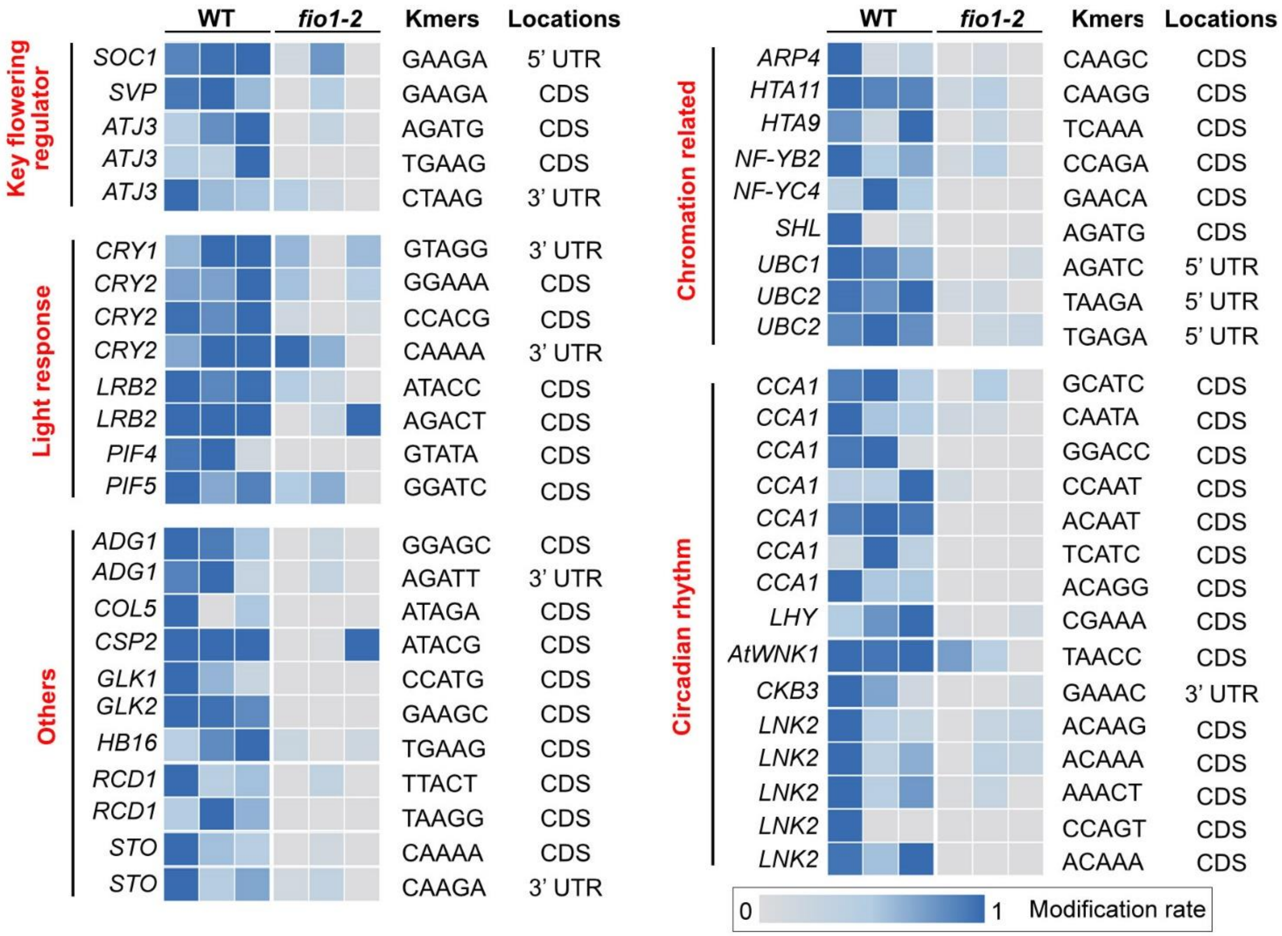

Figure S12. Heatmap showing the modification rates of flowering-related genes. The k-mers and positions of these hypomethlated sites are shown on the right. These genes are grouped based on their biological function in regualting flowering. *SOC1*, *SUPPRESSOR OF OVEREXPRESSION OF CONSTANS 1*; *SVP*, *SHORT VEGETATIVE PHASE*; *ARP4*, *ACTIN-RELATED PROTEIN 4*; *HTA11*, *HISTONE H2A 11*; *SHL*, *SHORT LIFE*; *NF-YB4*, *NUCLEAR FACTOR Y, SUBUNIT B4*; *UBC1*, *UBIQUITIN-CONJUGATING ENZYME 1*; *CCA1*, *CIRCADIAN CLOCK ASSOCIATED 1*; *LHY*, *LATE ELONGATED HYPOCOTYL*; *AtWNK1*, *ARABIDOPSIS THALIANA WITH NO LYSINE (K) KINASE 1*; *CKB3*, *CASEIN KINASE II BETA CHAIN 3*; *LNK2*; *NIGHT LIGHT-INDUCIBLE AND CLOCK-REGULATED GENE 2*; *CRY1*, *CRYPTOCHROME 1*; *LRB2*, *LIGHT-RESPONSE BTB 2*; *PIF4*, *PHYTOCHROME INTERACTING FACTOR 4*; *ADG1*, *ADP GLUCOSE PYROPHOSPHORYLASE 1*; *COL5*, *CONSTANS-LIKE 5*; *CSP2*, *COLD SHOCK PROTEIN 2*; *GLK1*, *GOLDEN2-LIKE 1*; *HB16*, *HOMEOBOX PROTEIN 16*; *RCD1*, *RADICAL-INDUCED CELL DEATH1*; *STO*, *SALT TOLERANCE*.

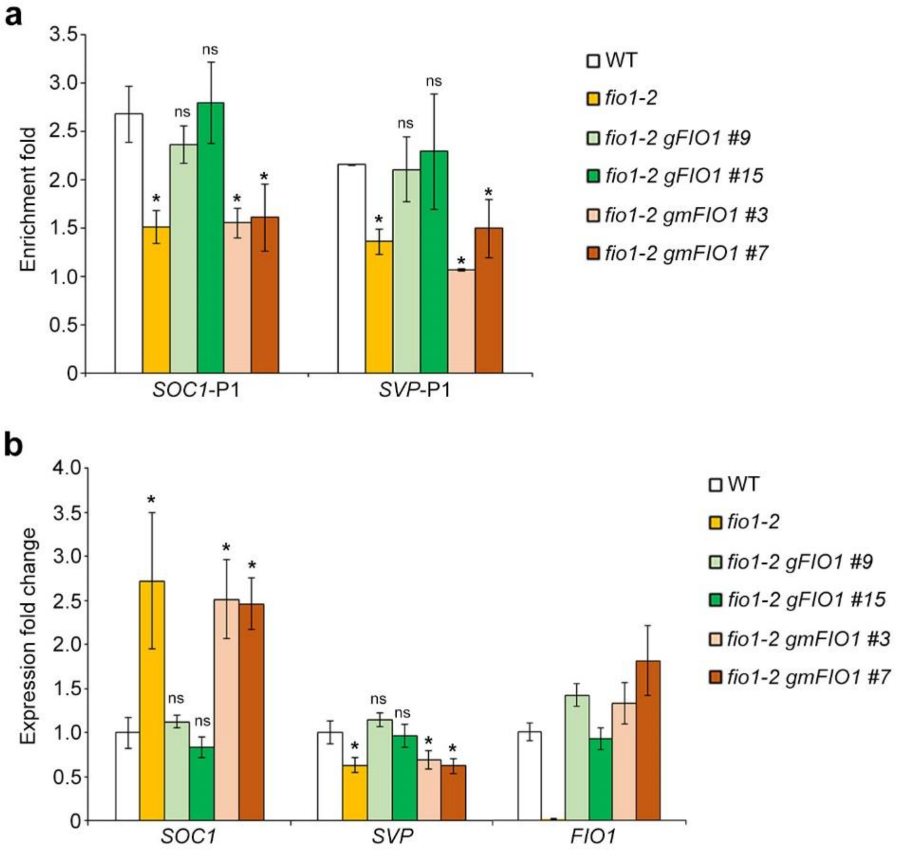

Figure S13. Analysis of m^{6}A enrichment and expression of *SOC1* and *SVP* in *fio1-2 gFIO* and *fio1-2 gmFIO1* transgenic plants. a) Analysis of m^{6}A enrichment on *SOC1* and *SVP* transcripts in various genetic background. m^{6}A-IP-qPCR was performed with 6-day-old seedlings in different genetic backgrounds under LDs. Error bars, mean ± SD; n = 3 biological replicates. Asterisks or ns indicate statistically significant differences or no statistical difference in m^{6}A enrichment levels between the indicated genotypes and wild-type seedlings (two-tailed paired Student's *t*-test, *$P < 0.05$; ns, $P > 0.05$). b) Quantitative analysis of expression levels of *SOC1*, *SVP*, and *FIO1* in various genetic backgrounds. Six-day-old seedlings grown under LDs were harvested for expression analysis. The expression levels were normalized to *TUB2* expression and then normalized to the expression level of each gene in wild-type set as 1.0. Error bars, mean ± SD; n = 3 biological replicates. Asterisks or ns indicate statistically significant differences or no statistical difference in expression levels between the indicated genotypes and wild-type seedlings (two-tailed paired Student's *t*-test, *$P < 0.05$; ns, $P > 0.05$).

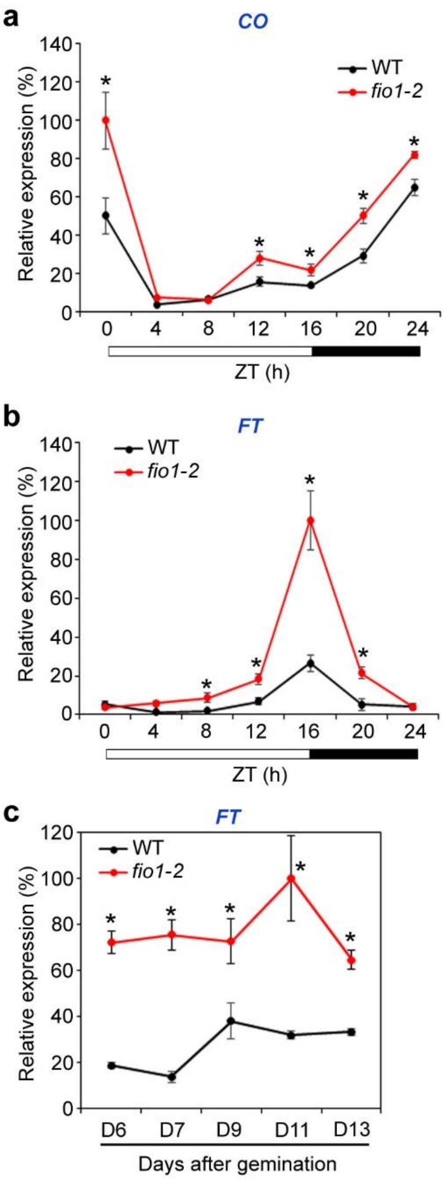

Figure S14. Expression of *CO* and *FT* in *fio1-2* mutants. a,b) Diurnal oscillation of *CO* (a) and *FT* (b) expression determined by real-time PCR in 6-day-old wild-type and *fio1-2* seedlings under LDs. Gene expression levels were normalized to *TUB2* expression, and the maximal expression level in each panel was set as 100%. Error bars, mean ± SD; n = 3. c) Temporal expression of *FT* determined by real-time PCR in developing wild-type and *fio1-2* seedlings under LDs. Gene expression levels were normalized to *TUB2* expression with the maximal expression level set as 100%. Error bars, mean ± SD; n = 3. Asterisks in (a-c) indicate significant differences between *fio1-2* and wild-type seedlings (two-tailed paired Student's *t*-test, $P < 0.05$).

a

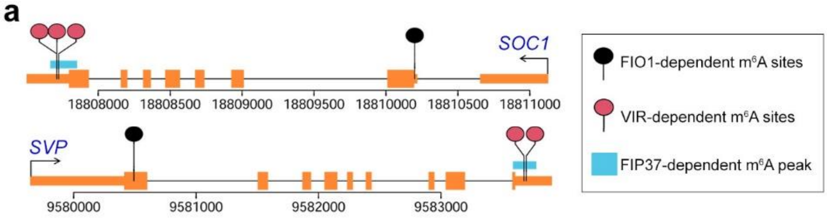

fip37-4 LEC1:FIP37 vs. WT (5-day-old seedlings)

Gene	Position	Location	m6A enrichment fold	
			WT	*fip37-4 LEC1:FIP37*
SOC1	18807668-18807854	3' UTR	8.46305	not detected
SVP	9583591-9583777	3' UTR	7.03386	not detected

vir-1 vs. *VIR*-complemented line (2-week-old seedlings)

Gene	Position	Location	m6A relative level	
			VIR-complemented line	*vir-1*
SOC1	18807712	3' UTR	1.0	0.36349
	18807713	3' UTR	1.0	0.66896
	18807725	3' UTR	1.0	0.09278
SVP	9583678	3' UTR	1.0	0.26609
	9583697	3' UTR	1.0	0.16724

fio1-2 vs. WT (6-day-old seedlings)

Gene	Position	Location	m6A relative level	
			WT	*fio1-2*
SOC1	18810200	5'UTR/CDS junction	1.0	0.32718
SVP	9580492	CDS	1.0	0.18085

b

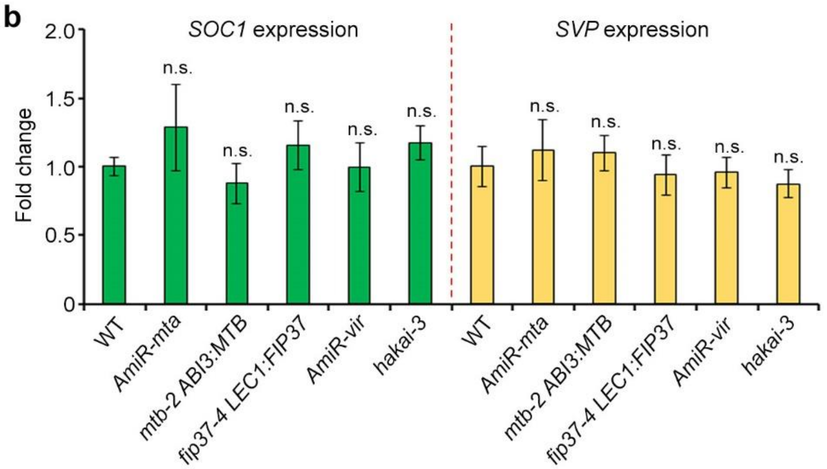

Figure S15. FIO1-mediated m^{6}A methylation modulates *SOC1* and *SVP* expression independently of other known m^{6}A writers. a) m^{6}A modification of *SOC1* and *SVP* affected by various m^{6}A writers. Schematic diagrams in the upper panels show the positions of FIO1-dependent m^{6}A sites, VIR-dependent m^{6}A sites and FIP37-dependent m^{6}A peak in *SOC1* and *SVP* transcripts. Thick and thin orange boxes represent exons and UTRs, respectively, and black lines represent introns. The following tables show the m^{6}A enrichment fold of *SOC1* and *SVP* in wild-type vs. *fip37-4 LEC1:FIP37* plants, and the m^{6}A relative levels of *SOC1* and *SVP* in *VIR*-complemented lines (*VIR::GFP-VIR*) vs. *vir-1* and wild-type vs. *fio1-2* plants. The m^{6}A-seq data

using 5-day-old wild-type and *fip37-4 LEC1:FIP37* seedlings[13] and the nanopore direct RNA sequencing data obtained from 2-week-old *VIR*-complemented line and *vir-1* seedlings[9] as well as 6-day-old wild-type and *fio1-2* seedlings in this study were used for the comparison. For nanopore sequencing data, the m^6A levels in *VIR*-complemented lines or wild-type plants were set as 1.0. b) Quantitative real-time PCR analysis of *SOC1* and *SVP* expression in 6-day-old seedlings in different genotypes. *AmiR-mta* and *fip37-4 LEC1:FIP37* were previously reported.[13] *mtb-2 ABI3:MTB* was generated by complementing the embryo lethality of *mtb-2* (CS850592) with the *ABI3:MTB* transgene, in which *MTB* was driven by the embryo-specific promoter of *ABA INSENSITIVE 3* (*ABI3*). The knockdown line *AmiR-vir* was generated by artificial microRNA (AmiR) interference, while *hakai-3* containing a 1-bp of guanine (G) deletion was generated by CRISPR/Cas9-mediated gene editing of the first exon of *HAKAI*. Results were normalized against the expression levels of *TUB2*, and the values in wild-type plants were set as 1.0. Error bars, mean ± SD; n = 3 biological replicates. n.s. indicate no significant difference between wild-type and other plants (two-tailed paired Student's *t* test, $P > 0.05$).

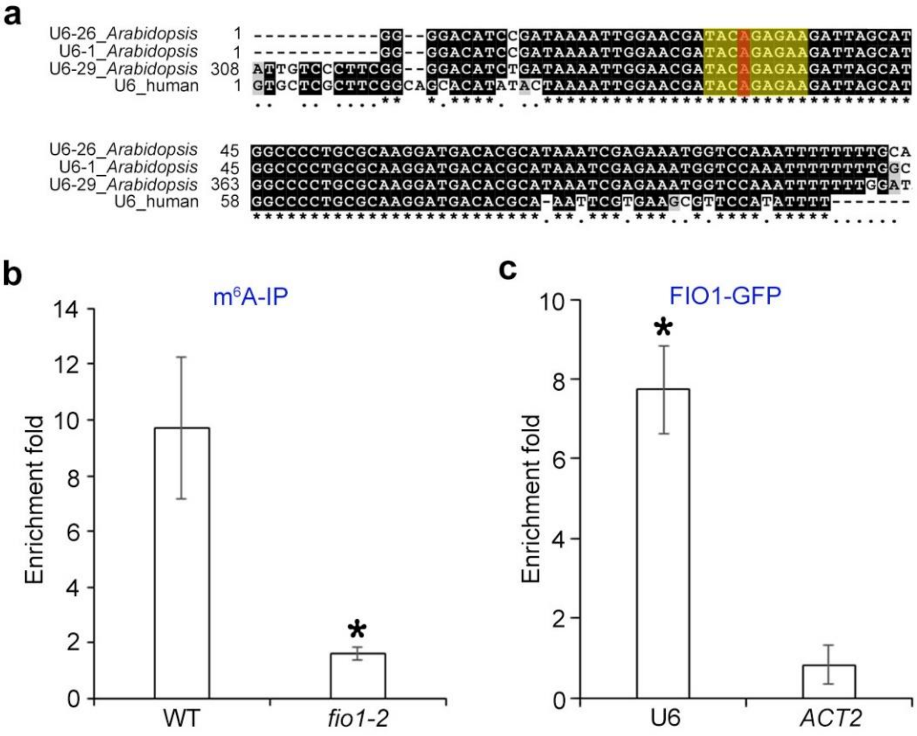

Figure S16. FIO1 methylates U6 snRNA in *Arabidopsis.* a) Alignment of the sequences of three U6 snRNAs genes in *Arabidopsis* and the U6 gene from human. The UACAGAGAA sequence required for METTL16 methylation is conserved and highlighted in yellow color with the methylated A highlighted in red. b) m^6A level on U6 snRNA is reduced in *fio1-2* mutants. m^6A-IP-qPCR was performed with total RNA extracted from 6-day-old wild-type and *fio1-2* seedlings under LDs. Error bars, mean ± SD; n = 3 biological replicates. Asterisk indicates a significant difference in m^6A enrichment levels between *fio1-2* and wild-type seedlings (two-tailed paired Student's *t*-test, $P < 0.05$); c) FIO1 directly binds to U6 snRNA. Six-day-old wild-type and *fio1-1 CsVMV:FIO1-GFP* seedlings grown under LDs were harvested for RNA immunoprecipitation assay. Error bars, mean ± SD; n = 3 biological replicates. Asterisk indicates a significant difference in FIO-GFP enrichment on U6 compared with the *ACTIN2* (*ACT2*) negative control (two-tailed paired Student's t-test, $P < 0.05$).

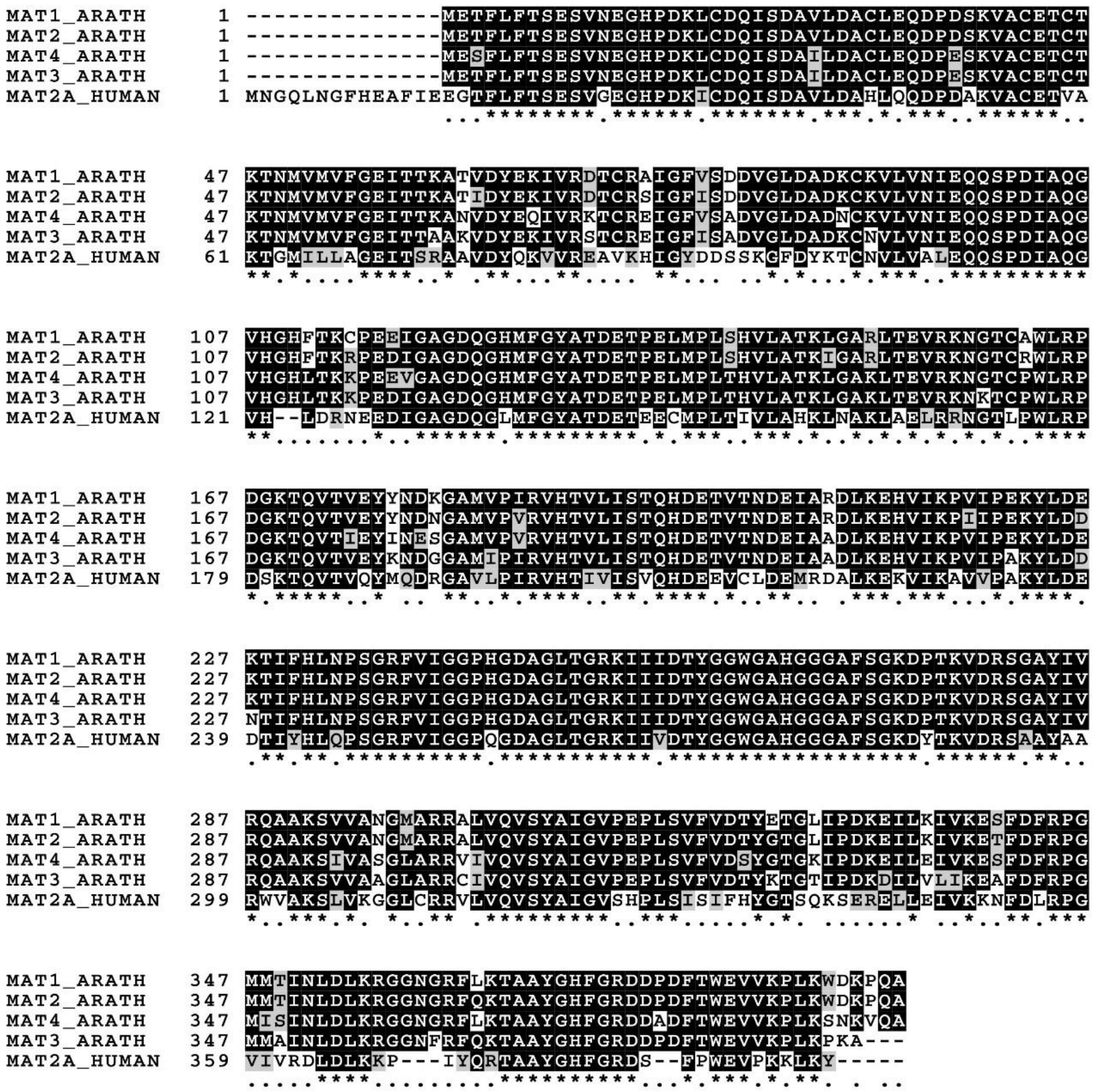

Figure S17. Protein sequence alignment of MAT1-4 from *Arabidopsis* and MAT2A from human. Amino acid sequences obtained from NCBI were aligned. Conserved residues are shown in black, while similar residues are shown in grey.

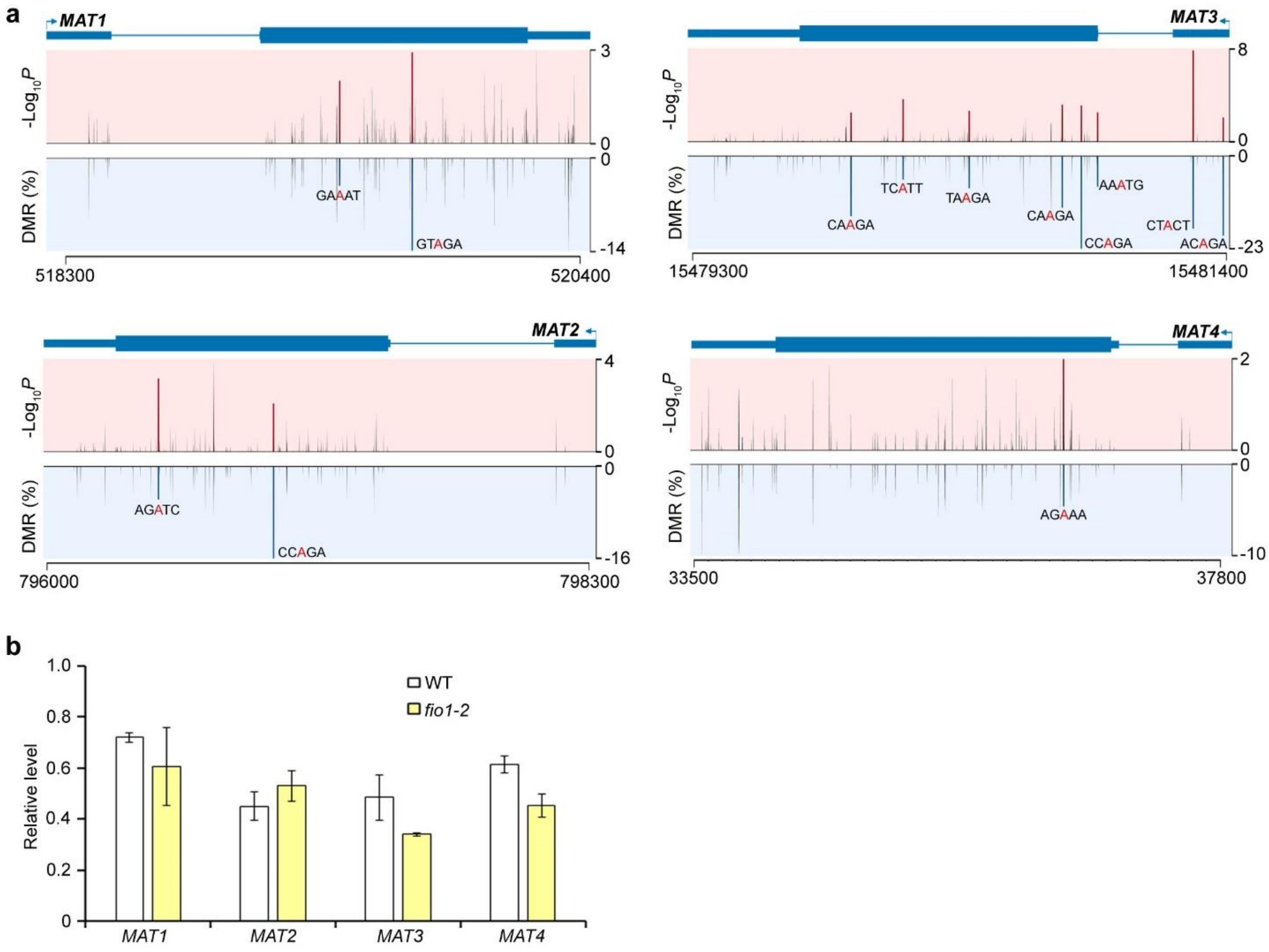

Figure S18. *MAT1-4* transcripts contain hypomethylated sites in *fio1-2*. a) Diagrams showing the DMRs, corresponding *P* values and the transcript sequences with identified m^6A sites of *MAT1-4* genes. The transcript structures are shown above. Thick and thin boxes represent exons and UTRs, respectively, while lines represent introns. b) Expression of *MAT1-4* in 6-day-old wild-type and *fio1-2* seedlings under LDs determined by real-time PCR. Error bars, mean ± SD; n = 3 biological replicates.
